# *S*-Glutathionylation of human inducible Hsp70 reveals a regulatory mechanism involving the C-terminal α-helical lid

**DOI:** 10.1074/jbc.RA119.012372

**Published:** 2020-04-24

**Authors:** Jie Yang, Hong Zhang, Weibin Gong, Zhenyan Liu, Huiwen Wu, Wanhui Hu, Xinxin Chen, Lei Wang, Si Wu, Chang Chen, Sarah Perrett

**Affiliations:** 1 National Laboratory of Biomacromolecules, CAS Center for Excellence in Biomacromolecules, Institute of Biophysics, Chinese Academy of Sciences, Chaoyang District, Beijing, China; 2 University of the Chinese Academy of Sciences, Shijingshan District, Beijing, China; 3 Beijing Institute for Brain Disorders, Youanmen, Beijing, China

**Keywords:** chaperone, oxidative stress, glutathionylation, 70-kilodalton heat shock protein (Hsp70), protein structure, cysteine modification, disulfide bond, HspA1A, protein homeostasis

## Abstract

Heat shock protein 70 (Hsp70) proteins are a family of ancient and conserved chaperones. Cysteine modifications have been widely detected among different Hsp70 family members *in vivo*, but their effects on Hsp70 structure and function are unclear. Here, we treated HeLa cells with diamide, which typically induces disulfide bond formation except in the presence of excess GSH, when glutathionylated cysteines predominate. We show that in these cells, HspA1A (hHsp70) undergoes reversible cysteine modifications, including glutathionylation, potentially at all five cysteine residues. *In vitro* experiments revealed that modification of cysteines in the nucleotide-binding domain of hHsp70 is prevented by nucleotide binding but that Cys-574 and Cys-603, located in the C-terminal α-helical lid of the substrate-binding domain, can undergo glutathionylation in both the presence and absence of nucleotide. We found that glutathionylation of these cysteine residues results in unfolding of the α-helical lid structure. The unfolded region mimics substrate by binding to and blocking the substrate-binding site, thereby promoting intrinsic ATPase activity and competing with binding of external substrates, including heat shock transcription factor 1 (Hsf1). Thus, post-translational modification can alter the structure and regulate the function of hHsp70.

## Introduction

Hsp70 proteins play an important role in maintaining protein homeostasis, including facilitating protein folding and degradation, preventing protein aggregation, and participating in the stress response (1). Due to interaction with numerous proteins, Hsp70 is involved in diverse physiological activities, such as signal transduction, apoptosis, transmembrane transport, and DNA repair (2–4). The number of members of the Hsp70 family present in each organism is observed to increase from bacteria to humans, reflecting the complex requirements of higher organisms. In *Escherichia coli*, three Hsp70 members have been found, whereas there are 14 members in *Saccharomyces cerevisiae* and 17 members in *Homo sapiens*, including HspA1A, which is the cytosolic stress-induced form (hHsp70), and HspA8, which is the cytosolic constitutively expressed form (hHsc70). If both *HSPA1A* and *HSPA8* genes are silenced by siRNA, the survival rate of cells is very low (5).

Structures available for Hsp70 homologues indicate two individual domains, namely the ATPase or nucleotide-binding domain (NBD) and the substrate-binding domain (SBD), connected by a flexible linker (6). The NBD contains two lobes (I and II), which can be further subdivided into four subdomains (IA, IB, IIA, and IIB) accommodating binding of ATP/ADP (7). The SBD is composed of a β-sheet-containing substrate-binding domain (SBDβ) and a C-terminal α-helical lid domain (SBDα) (8). SBDα has the lowest degree of sequence conservation among Hsp70 family members, but the structure, composed of four or five α-helixes, is generally conserved. The first helix, αA, rests against the SBDβ, whereas the remaining α-helices form an α-helical bundle, which acts as a lid over the substrate-binding site. Allosteric conformational changes in Hsp70 couple the ATP hydrolysis cycle in the NBD and the substrate-binding/release cycle in the SBD (9). The linker between the NBD and SBD facilitates allosteric conformational changes in the two domains (9, 10). Structural data for the *E. coli* Hsp70 homologue DnaK indicate that in the ATP-bound state, the NBD and SBD of Hsp70 are in a docked position, and substrate binds to the SBD in its SBDα lid-open state by relatively weak interactions that can promote ATP hydrolysis in the NBD (11–13). After ATP hydrolysis, the NBD is in the ADP-bound state, leading to undocking of the NBD and SBD, and strong interaction between substrate and the SBD in its SBDα lid-closed state (9). Nucleotide exchange factors (NEFs) promote exchange of ADP with ATP in the NBD, which then causes loosening of the interaction between substrate and the SBD and facilitates substrate release and exchange (9).

The functional cycle of Hsp70 can be regulated by a series of factors, including mutations, Hsp40 co-chaperones, NEFs, and tetratricopeptide repeat (TPR)-containing proteins. Hsp40 and NEFs interact with both the NBD and SBD of Hsp70, which promotes ATPase activity and substrate binding/release and accelerates the functional cycle of Hsp70 (9). The interaction of TPR proteins with other proteins enables them to act as adapter molecules in protein complexes (3). Binding of different TPR proteins to the EEVD motif in the C terminus of Hsp70 allows manifestation of the wide variety of Hsp70 functions, such as binding different substrates involved in diverse physiological activities within cells (3).

Post-translational modifications (PTMs) are an important means of functional regulation and signal transduction, and a number of PTMs have been identified in Hsp70, including phosphorylation (14), acetylation (15), ubiquitination (16), methylation (17), carboxylation (18), *S*-nitrosylation (19, 20), and *S*-glutathionylation (21–27). It is predicted that PTMs can also regulate the functional cycle of Hsp70 (28). Phosphorylation at Thr-36 of the yeast Hsp70 family member Ssa1 is found to affect interaction with certain substrates, leading to control of G_1_ cycling abundance and cell-cycle progression (14). Methylation at Lys-561 of hHsp70 or hHsc70 influences interaction of Hsp70 with substrates (17).

Thiols are nucleophilic and sensitive to the redox environment, allowing protein cysteine residues to undergo a broad range of redox modifications upon contact with reactive oxygen/nitrogen/sulfur species (ROS/RNS/RSS); these modifications include *S*-sulfenylation (-SOH), *S*-sulfinylation (-SO_2_H), *S*-sulfonylation (-SO_3_H), *S*-nitrosylation (-SNO), *S*-sulfhydration (-SSH), *S*-glutathionylation (-SSG), and inter-/intramolecular disulfide bond formation (-S-S-) (29, 30). Different cysteine modifications are often detected at the same site and frequently undergo further transformations (21, 30–32). Of these modifications, -SOH, -SNO, glutathionylation, and inter-/intramolecular disulfide bond formation are reversible, and unstable -SOH, -SNO, and thiyl radicals (-S^•^) can transform to the more stable glutathionylated form upon reaction with GSH (30–32). Exchange between glutathionylated and inter-/intramolecular disulfide forms also occurs (30). Thus, glutathionylation is a particularly important type of redox modification. Deglutathionylation is normally catalyzed by thioltransferase glutaredoxin (Grx) enzymes in the presence of GSH (32), although deglutathionylation of some proteins can occur spontaneously in the presence of GSH, so it is not fully dependent on Grx enzymes (26, 27, 33).

Glutathionylation is found to be involved in the basic functions of certain proteins (*e.g.* glutathionylation and deglutathionylation are essential for the functional cycle of β-tubulin and actin) as well as in free radical signal transduction (34, 35). Because glutathionylation is a reversible PTM, it can thus also protect proteins from undergoing irreversible oxidative modifications when subjected to oxidative stress; consequently, an increase in abundance of glutathionylated proteins is detected under oxidative conditions (34, 35). Glutathionylation, like phosphorylation, can also regulate cell structure, signal transduction, and metabolism through reversible modulation of the structure and function of specific proteins (34, 35). It has been shown that some chaperones are regulated by redox, including Hsp33, Asna1/TRC40, Hsp90, protein-disulfide isomerase, and Hsp27 (36). In addition, Hsp70 and Hsp60 are susceptible to glutathionylation under oxidative stress conditions (36). Glutathionylation of different members of the Hsp70 family has been detected in a variety of cells and tissues under oxidative conditions (21–27). Glutathionylation of hHsc70, the *E. coli* Hsp70 DnaK, and yeast ER-resident Hsp70 Kar2 regulates their chaperone activity (22, 26, 27, 37). However, the precise effects of glutathionylation on Hsp70 function and the mechanisms by which PTMs regulate function of Hsp70 family members are not clearly understood.

To help understand the physiological consequences of Hsp70 glutathionylation and the mechanism by which glutathionylation regulates the function of Hsp70, we investigated the susceptibility of hHsp70 to undergo glutathionylation both in cells and *in vitro* and then studied the effect of glutathionylation on the structure and function of hHsp70. From isolated SBDα to intact SBD and then to full-length Hsp70, we measured the effects on structure and function caused by glutathionylation within the SBDα and clearly observed how local structural changes induced by glutathionylation cause global effects on Hsp70 function. The NMR solution structure of the glutathionylated hHsp70 SBD reveals the structural basis for the altered peptide-binding affinity and ATPase activity upon glutathionylation.

## Results

### Detection of hHsp70 cysteine modification in HeLa cells

**Figure 1.**
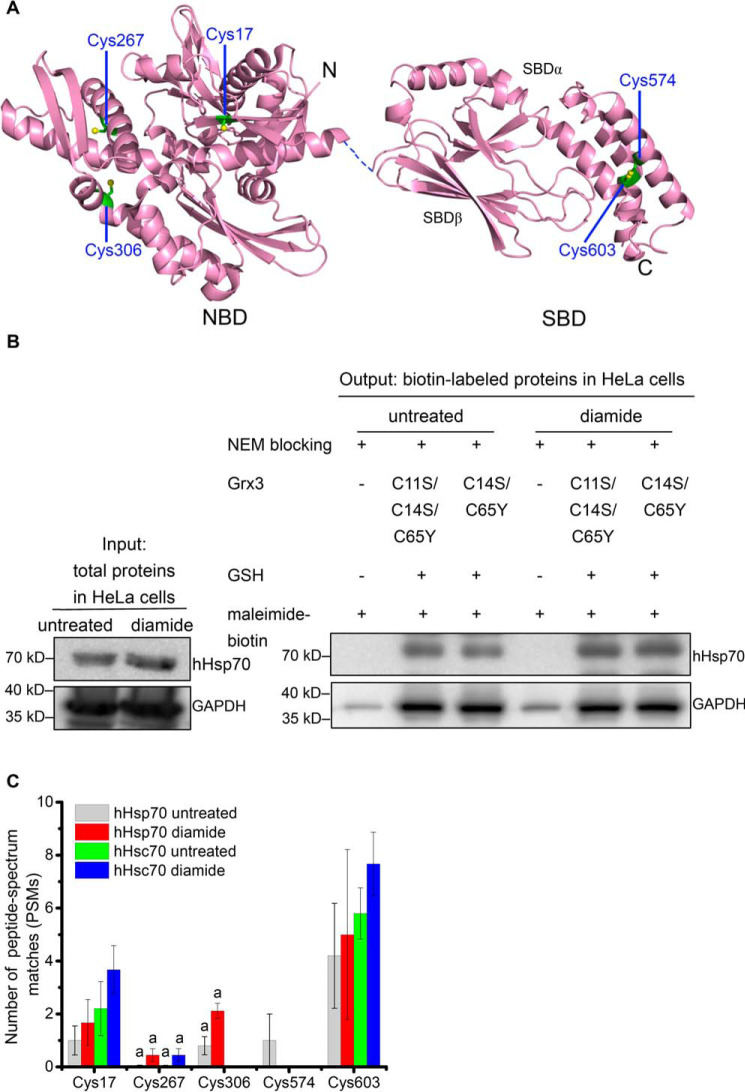
**Positions of Cys residues in hHsp70 and detection of hHsp70 glutathionylation in HeLa cells.** *A*, the crystal structures of the hHsp70 NBD in the ADP-bound state (PDB code 3AY9) and the SBD (PDB code 4PO2) are displayed in *purple*; the *dashed line* represents the flexible linker between the NBD and SBD; the β-sheet subdomain forming the substrate-binding site (SBDβ) and the α-helical subdomain (SBDα) that forms a lid over the substrate-binding site are indicated; the Cys residues (as indicated) are in *green. B*, representative Western blotting detection of glutathionylation of hHsp70 in HeLa cells with or without 1 mm diamide treatment consistent for three individual experiments. Before biotin switch, expression of hHsp70 and GAPDH in untreated and diamide-treated samples were compared. After blocking free cysteines present in the cell lysate mixture with NEM, untreated and diamide-treated samples were each divided into three aliquots: one without the reducing step with Grx and GSH, one reduced by Grx C11S/C14S/C65Y and GSH, and the third one reduced by Grx C14S/C65Y and GSH; each sample was then labeled with maleimide-biotin (biotin switch). Biotin-labeled proteins were enriched using streptavidin beads for Western blot analysis. Anti-hHsp70 mAb and anti-GAPDH polyclonal antibody were applied to detect hHsp70 and GAPDH in the enriched glutathionylated proteins, respectively. *C*, comparison of reversible cysteine modification of hHsp70 (HspA1) and hHsc70 (HspA8) in HeLa cells with or without diamide treatment detected by MS. After blocking free cysteines present in the cell lysate mixture with NEM, glutathionylated cysteines were reduced by Grx with GSH and labeled with maleimide-biotin (biotin switch) and then digested with trypsin. The biotin-labeled peptides were enriched for nanoLC-LTQ-Orbitrap XL MS/MS analysis. The data of untreated samples were averaged, and the S.E. (*error bars*) for five individual MS measurements (Spreadsheet S1, Data sets 1, 3, 4, 7, and 8) was calculated and likewise for the data of diamide-treated samples (three individual MS measurements, Spreadsheet S1, Data sets 2, 5, and 6). Peptides that could not be unambiguously assigned to a single Hsp70 family member are indicated with *a*, and in this case, the total number of PSMs was equally distributed among the possible source proteins.

Glutathionylation modifications have been reported in different Hsp70 family members in a variety of organisms, including four human Hsp70 family members (hHsc70, hHsp70, the ER-resident Hsp70 BiP, and the mitochondrial Hsp70 mortalin), *Chlamydomonas reinhardtii* HSP70B, yeast Kar2, and bacterial DnaK from both *E. coli* and *Salmonella typimurium* (21–27, 37). hHsp70 contains five Cys residues: Cys-17, Cys-267, and Cys-306, located in the NBD, and Cys-574 and Cys-603, located in the SBDα (Fig. 1*A*). However, the glutathionylation sites in hHsp70 have not yet been determined, although glutathionylation of hHsp70 has been detected previously by Western blotting (24). hHsc70, which has four Cys residues (Cys-17 and Cys-267 in the NBD and Cys-574 and Cys-603 in the SBD) has similarly been reported by other laboratories to undergo glutathionylation (21, 22), and glutathionylation at Cys-17 and Cys-603 has been detected in RAW264.7 mouse macrophages (33).

Here, we examined glutathionylation of hHsp70 in HeLa cells under both untreated and diamide-treated conditions. A strategy using resin-assisted enrichment of glutathionylated proteins or peptides after biotin switch is commonly used for detection of glutathionylation and other reversible cysteine modifications (27, 33, 38–41) and was also applied in this study. In brief, this strategy includes four steps: 1) irreversible blocking of unmodified free thiols with NEM, 2) reduction of reversibly modified thiols in the presence of GSH and Grx, 3) maleimide-biotin labeling of reduced free thiols, and 4) enrichment of biotin-labeled proteins for detection by Western blotting or enrichment of trypsin-digested peptides for MS. NEM blocking was performed throughout cell harvesting and cell lysis (*i.e.* at all steps prior to reduction by Grx and GSH) to prevent detection of false positives. To verify the blocking efficiency, we prepared samples without the Grx/GSH-reducing step for both untreated and diamide-treated conditions and found in both cases that biotin-labeled hHsp70 and GAPDH could not be detected or gave a very faint signal (Fig. 1*B*), confirming that the clear detection of biotin-labeled hHsp70 and GAPDH following the reducing step represents the occurrence of reversible cysteine modifications in cells (Fig. 1*B*). It is also clear that diamide treatment increased the degree of reversible cysteine modification of hHsp70 in HeLa cells, without any obvious change in hHsp70 expression level during the treatment time (Fig. 1*B* and Fig. S1). In contrast, for GAPDH, modification levels were similar with and without diamide treatment. GAPDH is a classic redox-regulated enzyme, and glutathionylation has been found to inhibit its enzymatic activity to transfer redox information to metabolic pathways (42, 43). In RAW264.7 mouse macrophages, diamide treatment was found to stimulate glutathionylation of GAPDH (33). The relatively oxidative environment of the HeLa cancer cell line may account for the high level of reversible cysteine modification of GAPDH even without diamide treatment. Grx generally shows promiscuous catalytic activity in reducing both glutathionylation and inter-/intramolecular disulfide bonds (44). Grx3 from *E. coli* with two mutations (C14S and C65Y) has highly specific activity to reduce glutathionylation (44), and Grx3 with three mutations (C11S, C14S, and C65Y), which no longer contains any cysteine residues, loses its reducing activity (27). Employing these mutants, we found that reduction of hHsp70 and GAPDH is not dependent on the reducing activity of Grx3 (Fig. 1*B*). Previous studies have also found that GSH allowed spontaneous full or partial reduction of reversible cysteine modifications of proteins (26, 27, 33).

The combined results of three independent rounds of cell sample preparation followed by biotin switch and MS indicate that Hsp70 family members HspA1A (hHsp70), HspA1L, HspA2, HspA4, HspA4L, HspA6, HspA7, HspA8 (hHsc70), HspA9, and HspA14 can each undergo reversible cysteine modifications in HeLa cells, generally at multiple sites (Fig. S2 and Spreadsheet S1, Data sets 1–8). Reversible cysteine modifications were detected in both untreated and diamide-treated cells, but the number of peptides detected tended to be higher in diamide-treated cells, as expected (Fig. S2 and Spreadsheet S1, Data sets 1–8). We tested both 1 and 10 mm diamide and found that whereas 10 mm diamide induced a higher level of cell death, for the surviving cells, the reversible cysteine modification levels of Hsp70 proteins were similar for the different concentrations of diamide (Spreadsheet S1, Data sets 2, 5, and 6). Reversible cysteine modification can potentially occur at all five cysteine residue positions in hHsp70 (Fig. 1*C* and Spreadsheet S1, Data sets 1–8). However, peptides that could be uniquely assigned to hHsp70 included Cys-17, Cys-574, and Cys-603, whereas peptides containing Cys-267 and Cys-306 could not be unambiguously assigned to a single Hsp70 family member (Fig. 1*C* and Table 1). For the four potential reversible cysteine modification sites in hHsc70 (HspA8), unique peptides included Cys-17 and Cys-603, whereas the peptides containing Cys-267 could not be unambiguously assigned, and Cys-574 was not detected (Fig. 1*C* and Table 1). Of reversible cysteine modifications that can occur, glutathionylation is the most likely to be detected due to its relative stability, and other types of reversible cysteine modifications can transform further into the glutathionylated form (30). Diamide treatment generally induces disulfide bond formation, but in the presence of excess GSH, the glutathionylated form (rather than intra-/intermolecular protein disulfide bonds) will predominate (45). Therefore, further *in vitro* characterization of hHsp70 was then carried out to ascertain the reactivity of its cysteine residues and the effect of glutathionylation on the structure and function of hHsp70.

**Table 1:**
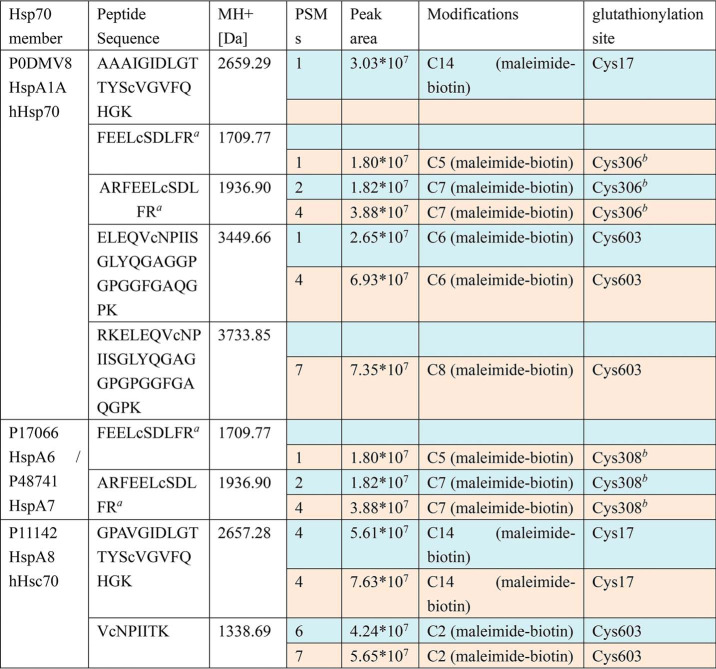
**Representative results from one round of HeLa cell preparation followed by biotin switch enrichment and nanoLC-LTQ-Orbitrap XL MS/MS detection, showing identified glutathionylation sites on different Hsp70 family members in untreated (*blue rows*) and diamide-treated cells (*beige rows*)**

*^a^* Source data have been deposited into the ProteomeXchange Consortium via the PRIDE partner repository with the data set identifier PXD017717. Spreadsheet S1 contains MS data sets for human Hsp70 (HspA) glutathionylation as follows: Data set 1, sample 1 untreated (*blue rows* in Table 1); Data set 2, sample 1 treated with 10 mm diamide (*beige rows* in Table 1); Data set 3, sample 2 untreated (MS detection 1 of 2); Data set 4, sample 2 untreated (MS detection 2 of 2); Data set 5, sample 2 treated with 1 mm diamide (MS detection 1 of 2); Data set 6, sample 2 treated with 1 mm diamide (MS detection 2 of 2); Data set 7, sample 3 untreated (MS detection 1 of 2); Data set 8, sample 3 untreated (MS detection 2 of 2).

*^b^* Peptide is found in both hHsp70 (HspA1A) and in HspA6/A7, so it cannot be unambiguously assigned.

### Characterization of hHsp70 glutathionylation propensity

**Figure 2.**
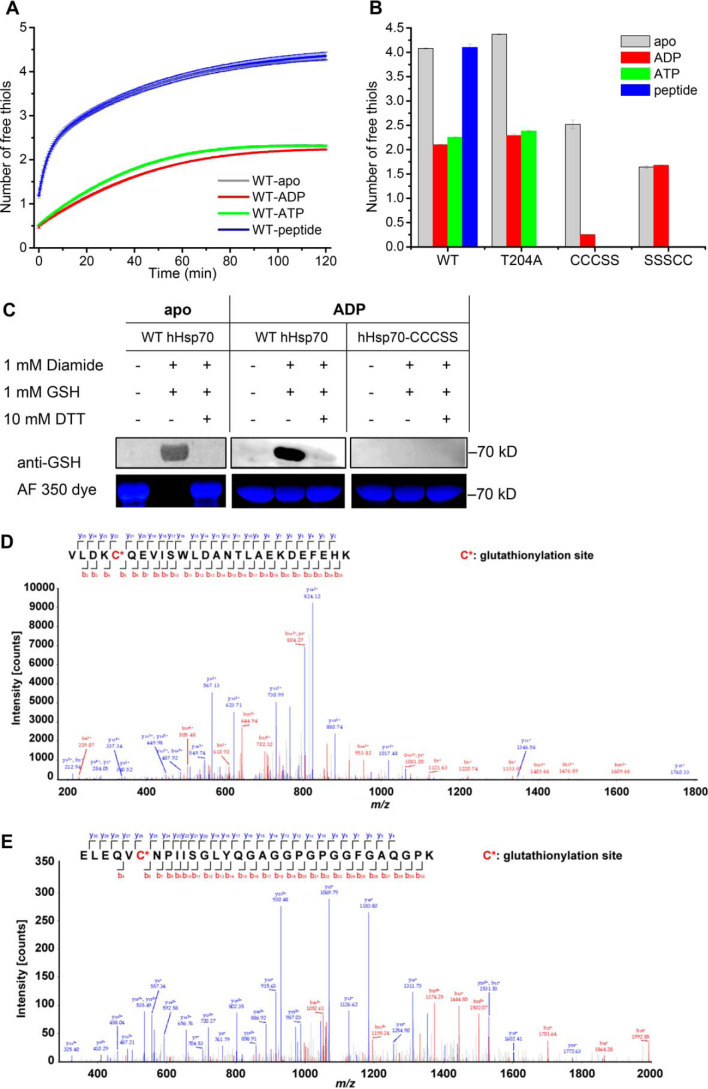
**Characterization of glutathionylation within the SBDα lid of full-length hHsp70 using purified hHsp70.** *A*, the time course of cysteine reactivity of hHsp70 in the absence and presence of ADP, ATP, or peptide was monitored in a Fluostar BMG plate reader, and the number of free thiols was calculated. The data shown are the mean of three independent measurements (each of three replicates), and the *error bars* represent the S.E. *B*, the number of free thiols measured at the 60-min time point is plotted to allow comparison of cysteine reactivity of WT hHsp70 and its mutants hHsp70 T204A, hHsp70-CCCSS, and hHsp70-SSSCC in the absence and presence of ADP, ATP, or peptide. Other details are as for *A. C*, Western blotting detection and Alexa Fluor® 350 dye staining distinguish glutathionylation and nonglutathionylation of WT hHsp70 in the absence of nucleotide, and WT hHsp70 or hHsp70-CCCSS in the presence of 1 mm ADP; 1:500 anti-GSH was used in the Western blotting detection. *D* and *E*, detection by MS of glutathionylation at Cys-574 (*D*) and Cys-603 (*E*) of purified WT hHsp70 treated with diamide and GSH in the presence of ADP. NanoLC-LTQ-Orbitrap XL MS/MS analysis confirmed the 3365.6-Da glutathionylated peptide VLDKC*^574^QEVISWLDANTLAEKDEFEHK (*D*) and the 3303.5-Da glutathionylated peptide ELEQVC*^603^NPIISGLYQGAGGPGPGGFGAQGPK (*E*) after trypsin digestion. The detected peaks (*main panel*) correspond to the predicted peptides (*inset*), where *red* corresponds to observed N-terminal peptide fragments and *blue* corresponds to observed C-terminal peptide fragments. C* indicates Cys-574 and Cys-603, which undergo glutathionylation.

Having established that Hsp70 can undergo reversible cysteine modifications such as glutathionylation in the cellular environment, we then carried out a thorough characterization of the effects of such modification on purified protein, to determine the potential structural and functional consequences of this PTM. To first test the susceptibility of hHsp70 to undergo cysteine modification, the reactivity of the cysteine residues in hHsp70 was evaluated by DTNB assay. In apo-hHsp70, the level of activity suggests four or five free thiols, and the presence of peptide substrate had no effect on the cysteine reactivity (Fig. 2, *A* and *B*). The presence of ATP or ADP decreased the cysteine reactivity of WT hHsp70 or its mutant T204A (which cannot hydrolyze ATP, analogous to the T199A mutation of DnaK (46)) to the equivalent of two free thiols (Fig. 2 (*A* and *B*) and Table 2). Upon binding of ADP, the cysteine reactivity of hHsp70-SSSCC (in which the three Cys residues in the NBD were mutated to Ser, leaving only the two Cys residues in the SBD) was unchanged, whereas the cysteine reactivity of hHsp70-CCCSS (which has only the three Cys residues in the NBD) was decreased (Fig. 2*B*). This indicates that the degree of cysteine reactivity detected in the presence of ATP or ADP can be attributed to the two C-terminal Cys residues. Thus, in the nucleotide-bound state of hHsp70, which is expected to be the predominant state *in vivo*, the Cys residues that are likely to be most susceptible to modification are Cys-574 and Cys-603, located in the αC and αD of the SBDα, respectively (Fig. 1*A*).

**Table 2:** **Summary of human HspA1A (hHsp70) mutants used in this study**

Name of protein	Description
WT hHsp70	hHsp70 (HspA1A/B)
hHsp70-SSSCC	hHsp70 C17S/C267S/C306S
hHsp70-CCCSS	hHsp70 C574S/C603S
hHsp70-CSSCC	hHsp70 C267S/C306S
hHsp70-CSSAA	hHsp70 C267S/C306S/C574A/C603A
hHsp70-CSSAS	hHsp70 C267S/C306S/C574A/C603S
hHsp70-CSSSS	hHsp70 C267S/C306S/C574S/C603S
hHsp70-CCCQQ	hHsp70 C574Q/C603Q, mimics glutathionylation of CTD
hHsp70-CSSCA	hHsp70 C267S/C306S/C603A
hHsp70-CSSAC	hHsp70 C267S/C306S/C574A
WT hHsc70	hHsc70 (HspA8)
SBDα(537–610) or SBDα(537–610)-CC	hHsp70 Δ1–536/Δ611–641, SBDα of hHsp70 consisting of remote part of α-helix B and α-helices C and D
SBDα(537–610)-AA	hHsp70 Δ1–536/Δ611–641/C574A/C603A
SBDα(537–610)-AS	hHsp70 Δ1–536/Δ611–641/C574A/C603S
SBDα(537–610)-SS	hHsp70 Δ1–536/Δ611–641/C574S/C603S
SBDα(537–610)-QQ	hHsp70 Δ1–536/Δ611–641/C574Q/C603Q
hHsp70(385–641)	hHsp70 Δ1–384, SBD of hHsp70
hHsp70 T204A	hHsp70 mutant that binds but does not hydrolyze ATP

We next investigated conditions that could replicate the glutathionylation reaction *in vitro*. Conditions that have been previously observed to cause glutathionylation of purified proteins include decomposed GSNO, fresh GSNO, GSSG, GSH and diamide, or GSH and H_2_O_2_ (22, 27, 47). We found that apo-hHsp70 was readily glutathionylated in the presence of decomposed GSNO, GSSG, GSH and diamide, or GSH and H_2_O_2_. Of these conditions, we found that GSH and diamide was the most efficient (Fig. S3), and this was therefore adopted as the standard method of glutathionylation for *in vitro* sample preparation, to obtain a homogeneous degree of glutathionylation, allowing rigorous structural and functional studies, comparing the properties of the glutathionylated and unmodified forms of the hHsp70 protein. Western blotting using anti-GSH antibody together with staining of free thiols with the maleimide-functionalized dye Alexa Fluor® 350 showed that all five cysteines in WT hHsp70 were glutathionylated in the apo state (Fig. 2*C*), whereas in the presence of ADP, WT hHsp70 was partially glutathionylated, and no glutathionylation occurred in the mutant hHsp70-CCCSS, in which the Cys residues within the SBD have been mutated to Ser (Fig. 2*C*), consistent with the results of thiol activity measurements (Fig. 2, *A* and *B*). NanoLC-LTQ-Orbitrap XL MS/MS of WT hHsp70 treated with diamide and GSH in the presence of ADP showed that peptides containing Cys-574 and Cys-603 were glutathionylated (Fig. 2, *D* and *E*), whereas no glutathionylated peptides containing Cys-17 or Cys-267 were detected. A small proportion of peptides containing Cys-306 were observed to be glutathionylated, possibly reflecting the strong MS signal of these peptides and occasional modification at Cys-306 in the presence of ADP. The results confirm that Cys-574 and Cys-603 undergo glutathionylation in ADP-bound WT hHsp70 *in vitro* (Fig. 2, *D* and *E*), consistent with the Western blotting and DTNB assay results above.

Combining the results of glutathionylation detection in cells and in purified protein, it is clear that Cys-574 and Cys-603 in the SBDα of hHsp70 have the highest propensities to undergo glutathionylation under physiological conditions, given the susceptibility of these two residues to undergo glutathionylation in both the presence and absence of nucleotide. Thus, further characterization was focused on the effects of glutathionylation within the SBD.

### Glutathionylation at Cys-574 and Cys-603 causes unfolding of the SBDα

**Figure 3.**
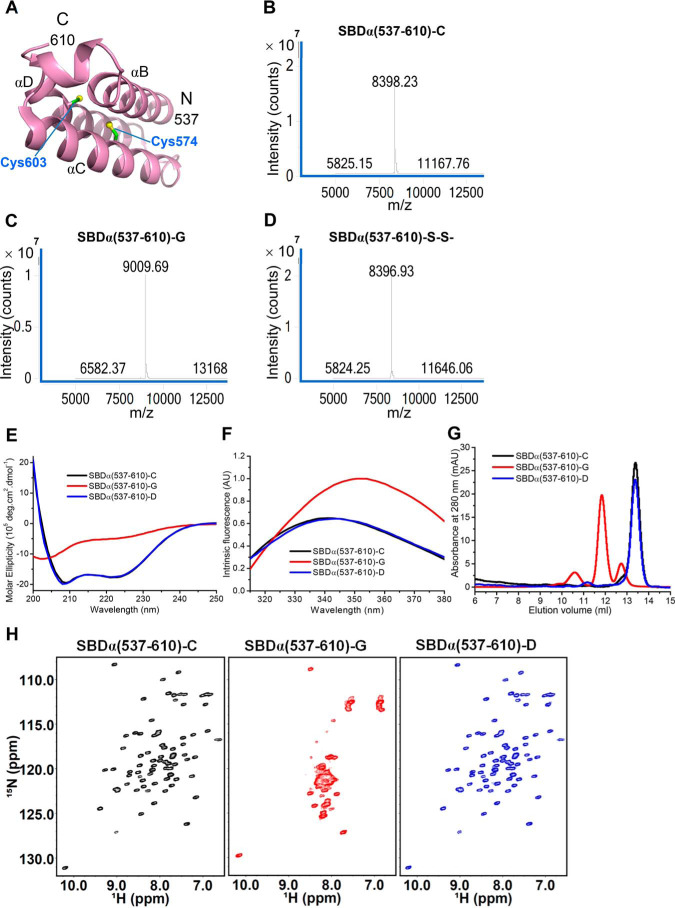
**Effect of glutathionylation at Cys-574 and Cys-603 on the structure of the hHsp70 SBDα(537–610).** *A*, structure of the hHsp70 SBDα(537–610) (PDB code 2LMG); Cys-574 and Cys-603 are in *green. B–D*, the molecular weight of SBDα(537–610) was analyzed by MS to detect glutathionylation (*-G*) or disulfide bond formation (*-S-S-*); shown before (*B*) and after (*C*) treatment with diamide and GSH, and after treatment with diamide alone (*D*). *E–G*, conformation and secondary structure of untreated control (*-C*, *black*), glutathionylated (*-G*, *red*), and deglutathionylated (*-D*, *blue*) SBDα(537–610) were compared by far-UV CD (*E*), intrinsic tryptophan fluorescence (after excitation at 295 nm) (*F*), and SEC (*G*). *H*, ^1^H-^15^N HSQC spectra of untreated control (*-C*, *black*), glutathionylated (*-G*, *red*), and deglutathionylated (*-D*, *blue*) SBDα(537–610) were compared.

To investigate the mechanism by which glutathionylation regulates hHsp70, we used the truncation mutant SBDα(537–610), which consists of the remote part of α-helix B and α-helices C and D, forming the C-terminal α-helix bundle part of the SBDα lid (Figs. 1*A* and 3*A*), to study the local structural changes caused by glutathionylation of the C-terminal pair of Cys residues, Cys-574 and Cys-603. Q-TOF MS confirmed that glutathionylation occurs within the SBDα(537–610), and the 611-Da molecular weight difference is consistent with glutathionylation at both Cys-574 and Cys-603 (Fig. 3, *B* and *C*). In the absence of GSH, diamide treatment causes intramolecular protein disulfide bond formation of SBDα(537–610), and the molecular weight of the product is 2 Da smaller than untreated SBDα(537–610) in Q-TOF MS detection (Fig. 3*D*). The far-UV CD spectra indicate that glutathionylation of the SBDα(537–610) causes a transition from α-helical to random coil structure (Fig. 3*E*). There is a single tryptophan residue (Trp-580) within the SBDα(537–610). The tryptophan fluorescence spectra also indicate that glutathionylation induces unfolding of the SBDα(537–610) (Fig. 3*F*), and analytical SEC indicates an increase in hydrodynamic volume (Fig. 3*G*). The ^1^H-^15^N HSQC NMR spectrum of the SBDα(537–610) shows well-dispersed peaks, indicating a well-folded structure, as reported previously (48), but upon glutathionylation, the peaks are narrowly dispersed with backbone ^1^H-^15^N signals ranging between 7.6 and 8.5 ppm in the hydrogen dimension (Fig. 3*H*), indicating that glutathionylated SBDα(537–610) has a disordered conformation. After deglutathionylation of the glutathionylated SBDα(537–610) by treatment with DTT, the properties measured by CD, intrinsic fluorescence, SEC, and NMR are essentially the same as the original untreated control (Fig. 3, *E–H*), indicating that the global conformational change caused by glutathionylation is fully reversible.

### Glutathionylation at Cys-574 and Cys-603 causes collapse of the unfolded SBDα into the SBDβ substrate-binding site

**Figure 4.**
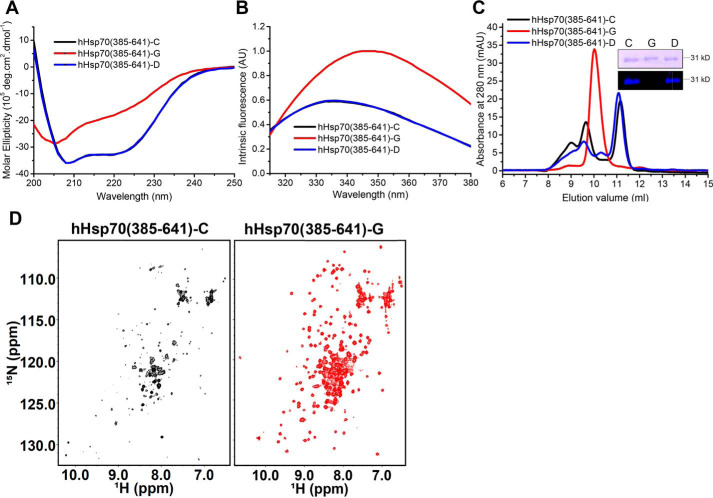
**Effect of glutathionylation at Cys-574 and Cys-603 on the structure of the hHsp70 SBD (*A–C*)**. Conformation and secondary structure of untreated control (*-C*, *black*), glutathionylated (*-G*, *red*), and deglutathionylated (*-D*, *blue*) hHsp70 SBD were compared by far-UV CD (*A*), intrinsic tryptophan fluorescence (after excitation at 295 nm) (*B*), and SEC (*C*). *Inset*, untreated control (*-C*), glutathionylated (*-G*), and deglutathionylated (*-D*) hHsp70 SBD were also examined by nonreducing SDS gel and Alexa Fluor® 350 dye staining. *D*, ^1^H-^15^N HSQC NMR spectra of untreated control (*-C*, *black*) and glutathionylated (*-G*, *red*) hHsp70 SBD were compared.

To further investigate the conformational changes that occur upon glutathionylation of the two C-terminal Cys residues, we used the truncation mutant hHsp70(385–641), henceforth referred to as the hHsp70 SBD, which contains both the SBDβ substrate-binding site and the SBDα (Fig. 1*A*). The far-UV CD spectra (Fig. 4*A*) and tryptophan fluorescence spectra (Fig. 4*B*) showed significant structural change consistent with loss of α-helical structure upon glutathionylation of the SBD, similar to the changes observed for the isolated SBDα(537–610) (Fig. 3, *E* and *F*), confirming that glutathionylation results in unfolding of the SBDα. Analytical SEC showed the disappearance of oligomeric elution peaks, indicating a reduction in oligomerization of the SBD upon glutathionylation (Fig. 4*C*). Similar to the observations for the isolated SBDα, the glutathionylated SBD is eluted earlier than unmodified SBD due to the increase in hydrodynamic volume caused by unfolding of the SBDα upon glutathionylation. The ^1^H-^15^N HSQC NMR spectrum of the hHsp70 SBD shows a number of weak peaks, consistent with a distribution of oligomeric states (Fig. 4*D*). Additionally, strong peaks are observed between 6.8 and 8.5 ppm in the ^1^H dimension, which can be attributed to backbone ^1^H-^15^N signals of residues in a random coil conformation as well as side chains of asparagine (7.8–8.5 ppm), glutamine, and arginine (6.8–7.8 ppm) residues. Upon glutathionylation, the spectrum becomes more homogeneous: the weak peaks become significantly stronger, and new peaks appear (Fig. 4*D*), indicating a transition from oligomer to monomer, consistent with the analytical SEC results (Fig. 4*C*). Comparison of the properties of untreated control and deglutathionylated SBD by CD, intrinsic fluorescence, and SEC again indicates that the structural changes induced by glutathionylation are fully reversible, and the SBDα(537–610) refolds upon deglutathionylation.

**Figure 5.**
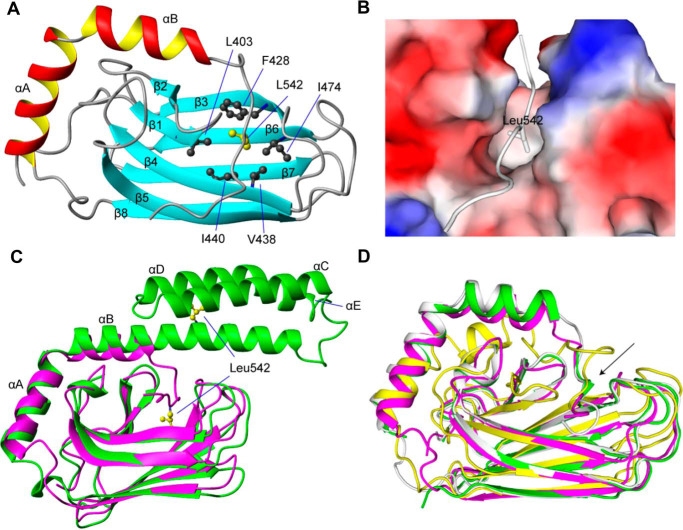
**The NMR structure of glutathionylated hHsp70 SBD.** *A*, *ribbon representation* of the glutathionylated hHsp70 SBD. Residues 611–641, which are disordered in the unmodified native structure, are not displayed. *B*, molecular surface of the glutathionylated hHsp70 SBD and binding of residue Leu-542 of the unraveled α-helix B within the hydrophobic core. *C*, superimposition of glutathionylated hHsp70 SBD (residues 385–641; *purple*) and nonglutathionylated hHsp70 SBD (residues 385–616, PDB code 4PO2; *green*). *D*, superimposition of glutathionylated hHsp70 SBD (residues 385–641; *magenta*) and truncated Hsp70 SBD domains of different species: rat Hsp70 (residues 383–542, PDB code 1CKR; *yellow*), human Hsp70 (residues 393–543, PDB code 4WV5; *green*), and bovine Hsp70 (residues 1–554, PDB code 1YUW; *white*). The *arrow* indicates the segments of the SBDα that have collapsed into the SBDβ.

To fully understand the structural basis for the functional changes induced by glutathionylation, we determined the structure of the glutathionylated hHsp70 SBD using solution NMR (Fig. 5, Fig. S4, and Table 3). The structure shows that the C-terminal α-helical bundle is completely disrupted and becomes disordered after glutathionylation at Cys-574 and Cys-603 (Fig. S4*B*). In the structure, the secondary elements β1–β8, αA, and part of αB (residues 524–535) are well-preserved, whereas αB (residues 536–545) and αC-αE, which were in an α-helical conformation in the unmodified state, are now in a loop conformation (Fig. 5, *A–C*). Notably, the side-chain methyl groups of residue Leu-542 bind in the hydrophobic cleft of the SBDβ, and thus the unfolded C-terminal region blocks the substrate-binding site (Fig. 5, *B* and *C*). Unfolded SBDα increases the hydrodynamic radius of monomeric SBD, whereas binding of the unfolded SBDα to the SBDβ decreases oligomerization of the SBD by blocking interactions mediated by the hydrophobic site of the SBDβ, also explaining the changes in SEC profile and signal enhancement of NMR peaks upon glutathionylation (Fig. 4, *C* and *D*). Structures of Hsp70 containing truncations of the SBDα also show a similar collapse of the unfolded C terminus into the substrate-binding site, including human, rat, and bovine homologues (49–51) (Fig. 5*D*), indicating that disruption of the structure of the α-helical lid will generally lead to blocking of the SBDβ substrate-binding site, due to binding of the unfolded C-terminal region in place of extrinsic substrates.

**Table 3:** **Experimental restraints and structural statistics for the 20 lowest-energy structures of glutathionylated hHsp70 SBD (residues 386–641)**

**Distance restraints**	
Intraresidue	866
Sequential	477
Medium	158
Long-range	451
Ambiguous	1249
Total	3201
Hydrogen bond restraints	90
**Dihedral angle restraints**	
ϕ	99
y	95
χ1	63
Total	257
**Violations**	
NOE violations (>0.3 Å)	0
Torsion angle violation (>5°)	0
**PROCHECK statistics (%)***^a^*	
Most favored regions	86.8
Additional allowed regions	11.4
Generously allowed regions	0.8
Disallowed regions	0.9
**r.m.s.d. from mean structure (Å)**	
Backbone heavy atoms	
All residues*^b^*	1.67 ± 0.28
Regular secondary structure*^c^*	0.42 ± 0.09
All heavy atoms	
All residues	2.37 ± 0.32
Regular secondary structure	1.22 ± 0.15

*^a^* Residues used to calculate PROCHECK statistics include 394–545.

*^b^* Residues used to calculate the r.m.s.d. values of all residues include 394–545 in glutathionylated hHsp70(385–641).

*^c^* Regular secondary structure regions used here include β-strand residues: 401–404, 410–415, 422–428, 438–444, 457–460, 474–480, 486–491, and 498–504 for glutathionylated hHsp70(385–641).

### Glutathionylation at Cys-574 and Cys-603 causes significant changes in structure and function of full-length hHsp70

**Figure 6.**
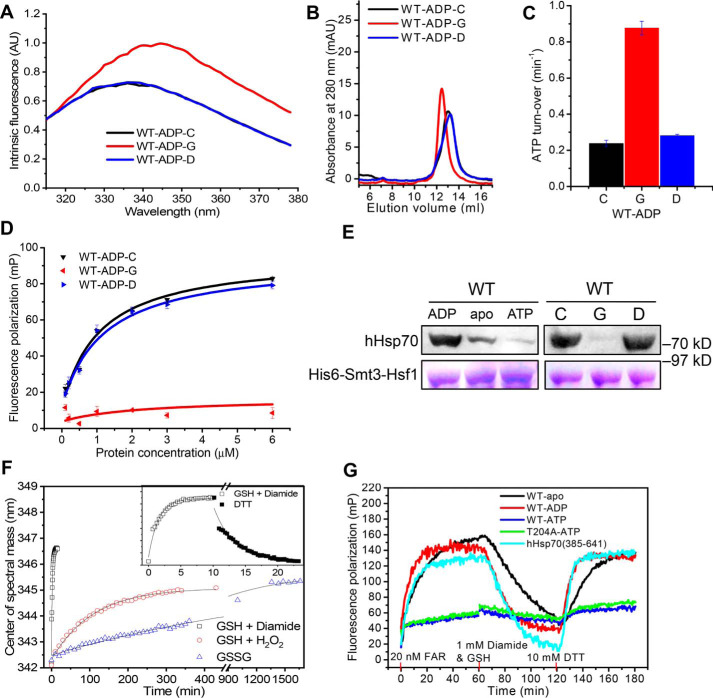
**Effect of glutathionylation at Cys-574 and Cys-603 on conformation and function of full-length hHsp70.** *A* and *B*, the conformation and oligomeric state of untreated control (*-C*, *black*), glutathionylated (*-G*, *red*), and deglutathionylated (*-D*, *blue*) full-length hHsp70 (residues 1–641) in the presence of 0.5 mm ADP in Buffer B were analyzed by intrinsic tryptophan fluorescence (after excitation at 295 nm) (*A*) and SEC (*B*), respectively. *C* and *D*, the ATPase activity (*C*) and peptide-binding ability (*D*) of untreated control (*-C*, *black*), glutathionylated (*-G*, *red*), and deglutathionylated (*-D*, *blue*) full-length hHsp70 (residues 1–641) in the presence of 0.5 mm ADP in Buffer B were compared. FP at 520 nm after excitation at 485 nm was used to monitor the binding of 20 nm FAR peptide to different concentrations of hHsp70 or its mutants, as indicated. The data shown are the mean of three individual experiments, and the *error bars* represent the S.E. *E*, the interaction of hHsp70 with Hsf1 was detected by a pulldown assay. WT hHsp70 in the absence or in the presence of 1 mm ADP or ATP and untreated control (*-C*), glutathionylated (*-G*), or deglutathionylated (*-D*) ADP-bound WT hHsp70 samples (20 μm) were incubated with 2 μm His_6_-Smt3-Hsf1 at 8 °C for 60 min, as indicated. Elution with Buffer C (50 mm Tris-HCl, pH 7.5, 300 mm KCl, 5 mm MgCl_2_) containing 300 mm imidazole (from the nickel-Sepharose separation step) was detected by SDS-PAGE staining with Coomassie Brilliant Blue and by Western blotting with anti-hHsp70 antibody. *F*, the time course of conformational change accompanying glutathionylation and deglutathionylation of WT hHsp70 in the presence of 0.5 mm ADP was monitored by recording the shift in CSM of the intrinsic fluorescence spectrum. Glutathionylation kinetics induced by 1 mm diamide and 1 mm GSH (*black squares*), 1 mm GSH and 4 mm H_2_O_2_ (*red circles*), and 2 mm GSSG (*blue triangles*) were compared. *Inset*, kinetics of glutathionylation induced by 1 mm diamide and 1 mm GSH (*open squares*) and kinetics of deglutathionylation (*filled squares*) induced by 10 mm DTT. *G*, kinetics of FAR peptide binding to 10 μm apo WT hHsp70 (*black*), ADP-bound WT hHsp70 (*red*), ATP-bound WT hHsp70 (*blue*), ATP-bound hHsp70 T204A (*green*), and SBD of hHsp70 (residues 385–641, *cyan*) accompanying glutathionylation and deglutathionylation were monitored and compared. 20 nm FAR, 1 mm diamide with 1 mm GSH, and 10 mm DTT were added at 0, 60, and 120 min, respectively, to initiate peptide binding, glutathionylation, and deglutathionylation in turn.

To fully investigate the mechanism by which glutathionylation regulates hHsp70 function, we used full-length hHsp70 to study structural and functional changes of intact hHsp70 caused by glutathionylation of the C-terminal pair of Cys residues, Cys-574 and Cys-603. hHsp70 contains two tryptophan residues, Trp-90 and Trp-580. Monitoring of intrinsic tryptophan fluorescence upon glutathionylation of ADP-bound full-length hHsp70 (Fig. 6*A*) shows similar spectral changes as observed upon glutathionylation of the isolated SBDα(537–610) and the complete SBD (Figs. 3*F* and 4*B*), confirming that glutathionylation also causes unfolding of the SBDα in intact hHsp70. Analytical SEC indicates a small increase in hydrodynamic volume, consistent with a switch to random coil structure by unfolding of the SBDα (Fig. 6*B*). Functional regulation of hHsp70 by glutathionylation was evaluated by ATPase activity and peptide substrate-binding ability (Fig. 6, *C* and *D*). Elevated ATPase activity and decreased peptide-binding ability of glutathionylated hHsp70 could be explained by collapse of the unfolded SBDα into the SBD substrate-binding site, mimicking substrate binding. Although the FITC-labeled ALLLSAPRR (FAR) peptide has an affinity of 1–10 μm for hHsp70, glutathionylation-induced unfolding of the SBDα almost abolished binding of FAR to hHsp70 (Table 4 and Fig. 6*D*), indicating a strong interaction between the unfolded SBDα and the SBDβ substrate-binding site. This is consistent with the results of previous studies using SBDα truncation mutants of other Hsp70 homologues, where similarly, tight binding of the unfolded SBDα to the SBD of DnaK or rat Hsc70 could not be displaced by high concentrations of peptides (up to 10 mm), presumably due to the high effective concentration of the unfolded SBDα when binding is intramolecular (50, 52). Hsf1 is a protein substrate of hHsp70 (53). It was found that the presence of ADP favored interaction between hHsp70 and hHsf1, whereas ATP disfavored this interaction (Fig. 6*E*). In the presence of ADP, glutathionylation of hHsp70 dramatically weakened this interaction (Fig. 6*E*), consistent with the results using peptide substrate, indicating that glutathionylation within the SBDα of hHsp70 switches off its chaperone function. After deglutathionylation of the glutathionylated hHsp70 by treatment with DTT, the properties measured by intrinsic fluorescence, SEC, ATPase activity, and substrate binding are essentially the same as the original untreated control (Fig. 6, *A–E*), indicating that the structural and functional changes induced by glutathionylation are fully reversible in full-length hHsp70.

**Table 4:** **FAR peptide dissociation constants for hHsp70 and its mutants**

State	*K_d_*
	μ*m*
**WT ADP**	
Control	1.07 ± 0.25
Glutathionylated	ND*^a^*
Deglutathionylated	1.20 ± 0.33
**Apo**	
WT	1.12 ± 0.12
hHsp70-CSSCC	1.35 ± 0.23
hHsp70-CSSAA	1.19 ± 0.21
hHsp70-CSSAS	1.44 ± 0.54
hHsp70-CSSSS	ND
hHsp70-CCCQQ	ND

*^a^* ND, not determined.

Kinetics of glutathionylation and deglutathionylation of hHsp70 could be measured by monitoring the time course of structural and functional changes accompanied by corresponding changes in intrinsic fluorescence and peptide binding. By following the time course of the change in the center of spectral mass (CSM) of intrinsic fluorescence during the glutathionylation reaction (Fig. 6*F*), we can estimate the reaction rate of glutathionylation. By fitting the curves to a single exponential function (Fig. 6*F*), we calculated the apparent reaction rate constant (*k*) for glutathionylation of Cys-574 and Cys-603 in ADP-bound hHsp70 under different conditions: *k* (GSH + diamide) = (1.09 ± 0.05) × 10^−2^ s^−1^, *k* (GSH + H_2_O_2_) = (1.54 ± 0.06) × 10^−4^ s^−1^, and *k* (GSSG) = (2.32 ± 0.12) × 10^−5^ s^−1^. Thus, in the presence of GSH, diamide causes the glutathionylation of hHsp70 much more rapidly than GSH with H_2_O_2_, or GSSG alone. The *k* value for deglutathionylation of Cys-574 and Cys-603 in hHsp70 by DTT was (5.48 ± 0.06) × 10^−3^ s^−1^. In fact, the time taken to observe a CSM shift includes both the thiol reaction time and the time taken for the subsequent structural changes, so the observed *k* value that we calculate represents the lower limit of the actual thiol reaction rate constant. During the fluorescence polarization (FP) monitored time course of peptide binding, we added diamide and GSH to glutathionylate hHsp70 and then DTT to deglutathionylate hHsp70. We observed a shift in the equilibrium of peptide binding toward dissociation upon glutathionylation and restoration of peptide binding upon deglutathionylation in both the isolated SBD of hHsp70 (truncation mutant hHsp70(385–641)) and full-length hHsp70, regardless of whether hHsp70 was in the apo, ADP-bound, or ATP-bound state (Fig. 6*G*). This suggests that the glutathionylation/deglutathionylation cycle of hHsp70 could potentially regulate substrate binding at any point in the hHsp70 functional cycle. The mutant T204A, which has extremely weak ATP hydrolysis ability, was used to avoid hydrolysis of bound ATP, and the result confirms that the regulation of substrate binding by glutathionylation and deglutathionylation also occurs in the ATP state of hHsp70 (Fig. 6*G*).

### Different contributions of glutathionylation at Cys-574 and Cys-603 to modulation of hHsp70

**Figure 7.**
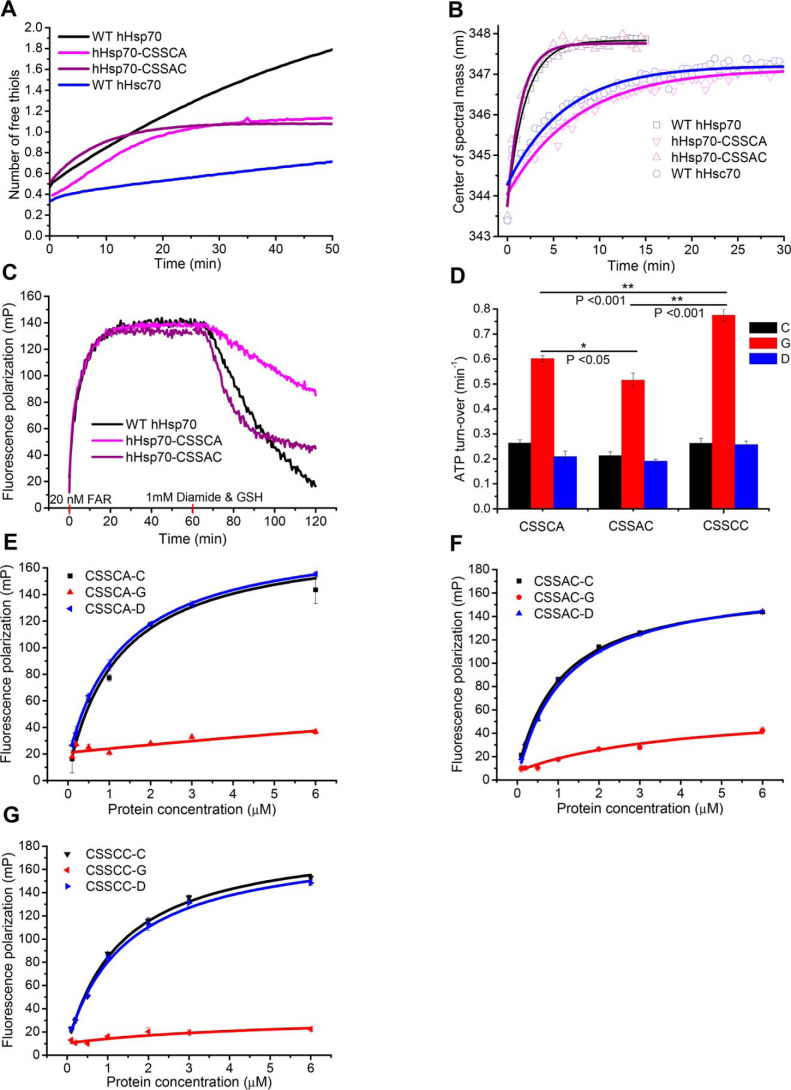
**Characterization of the relative susceptibility of residues Cys-574 and Cys-603 to undergo glutathionylation.** *A*, time course of cysteine reactivity of WT hHsp70 (*black*), hHsp70-CSSCA (*magenta*), hHsp70-CSSAC (*purple*), and WT hHsc70 (*blue*) in the presence of 0. 5 mm ADP were compared. The data shown are the mean of three independent measurements (each of three replicates). *B*, time courses of conformational change accompanying glutathionylation of WT hHsp70 (*black squares*), hHsp70-CSSCA (*magenta triangles*), hHsp70-CSSAC (*purple triangles*), and WT hHsc70 (*blue circles*) in the presence of 0.5 mm ADP were compared by monitoring CSM of the intrinsic fluorescence spectrum. Glutathionylation kinetics were induced by 1 mm diamide and 1 mm GSH. *C*, kinetics of FAR peptide binding to 10 μm WT hHsp70 (*black*), hHsp70 CSSCA (*magenta*), and hHsp70 CSSAC (*purple*) in the presence of 0.5 mm ADP accompanying glutathionylation were monitored and compared; 20 nm FAR and 1 mm diamide with 1 mm GSH were added at 0 and 60 min, respectively, to initiate peptide binding and glutathionylation in turn. *D–G*, ATPase activity (*D*) and peptide binding ability (*E–G*) of untreated control (*-C*, *black*), glutathionylated (*-G*, *red*), and deglutathionylated (*-D*, *blue*) hHsp70-CSSCA, hHsp70-CSSAC, and hHsp70-CSSCC in the presence of 0.5 mm ADP in Buffer B were compared.

To check whether Cys-574 and Cys-603 have different thiol reactivity and different contributions to redox regulation of hHsp70, DTNB reaction kinetics and glutathionylation kinetics of individual cysteine residues were further studied. The reactivity of ADP-bound hHsp70-CSSCA and hHsp70-CSSAC corresponds only to Cys-574 and Cys-603, respectively. In the presence of ADP, the DTNB assay shows that Cys-603 reacts more rapidly than Cys-574 (Fig. 7*A*), suggesting that Cys-603 is more reactive. Similarly, comparing the glutathionylation reaction rate of hHsp70-CSSCA and hHsp70-CSSAC induced by GSH and diamide by monitoring the CSM shift, we also detected a more rapid rate of glutathionylation for Cys-603 than for Cys-574: *k* (hHsp70-CSSAC) = (1.12 ± 0.05) × 10^−2^ s^−1^, *k* (hHsp70-CSSCA) = (2.19 ± 0.14) × 10^−3^ s^−1^ (Fig. 7*B*). The similar *k* value for WT hHsp70 of (1.09 ± 0.05) × 10^−2^ s^−1^ to that for hHsp70-CSSAC indicates that glutathionylation within the C-terminal α-helical lid is initiated by glutathionylation of Cys-603. Consistent with this, when monitoring by FP, hHsp70-CSSAC and WT hHsp70 showed a more rapid shift of the peptide-binding equilibrium toward dissociation upon glutathionylation than hHsp70-CSSCA (Fig. 7*C*). However, glutathionylation has a more profound effect on the ATPase activity and peptide-binding ability of hHsp70-CSSCC than of hHsp70-CSSCA or hHsp70-CSSAC (Fig. 7, *C–G*), indicating that modification at both Cys-574 and Cys-603 has a more significant effect on the structure and function of hHsp70 than modification at only one of these residues.

These results together indicate that Cys-574 and Cys-603 work differently and cooperate to regulate function of hHsp70 upon thiol modification, as follows: 1) Cys-603 has higher sensitivity to oxidative stress but a weaker effect on hHsp70 structure and function; 2) Cys-574 has lower sensitivity to oxidative stress, but glutathionylation of Cys-574 in combination with glutathionylation of Cys-603 has a stronger effect on hHsp70; 3) structural changes caused by modification of Cys-603 cause exposure of Cys-574, and thus modification of Cys-574 can be accelerated by modification of Cys-603. It is possible that mild oxidation of short duration in cells may only cause glutathionylation of Cys-603, inducing moderate structural and functional changes in hHsp70, whereas prolonged severe oxidation in cells may cause glutathionylation of both Cys-603 and Cys-574 and result in dramatic structural and functional changes of hHsp70. This could explain why glutathionylated Cys-603 is more frequently detected in cells by MS than glutathionylated Cys-574 (Table 1 and Fig. 1*C*). The different contributions of Cys-574 and Cys-603 to regulation caused by thiol modification may be determined by their position in the structure and the different contributions of Cys-574 and Cys-603 to the structural stability of the SBDα. Cys-574 is located in the center of αC, is fully buried, and contributes significantly to the stability of the hydrophobic core of the SBDα structure. Cys-603 is also well-buried, but it is located at the junction of αD and αE and so may more readily become accessible to modification due to “breathing” of the helix-bundle structure. Glutathionylation of the constitutively expressed Hsp70 homologue hHsc70 had been detected in different cell lines by different laboratories (21), including in this study (Fig. 1*C* and Table 1). hHsc70 has 86% sequence identity with hHsp70, including four cysteine and two tryptophan residues at the equivalent sites, but hHsc70 lacks the NBD residue Cys-306, which is present in hHsp70. The SBD residue Cys-603 in hHsc70 was also detected as being glutathionylated in HeLa cells. The DTNB assay showed that in the presence of ADP, hHsc70 has much lower thiol reactivity than hHsp70 (Fig. 7*A*). Comparing the glutathionylation reaction rate induced by GSH and diamide by monitoring the CSM shift, we also detected a lower rate of glutathionylation for hHsc70 than for hHsp70: *k* (hHsc70) = (2.67 ± 0.19) × 10^−2^ s^−1^. The *k* (hHsc70) is similar to *k* (hHsp70-CSSCA). This suggests that hHsp70 is more sensitive to redox than hHsc70 in cells, and Cys-603 of hHsp70 is the most sensitive among all Cys residues in hHsp70 in the nucleotide-bound state.

### Mutation of Cys-574 and Cys-603 causes structural and functional changes in hHsp70 mimicking those induced by glutathionylation

**Figure 8.**
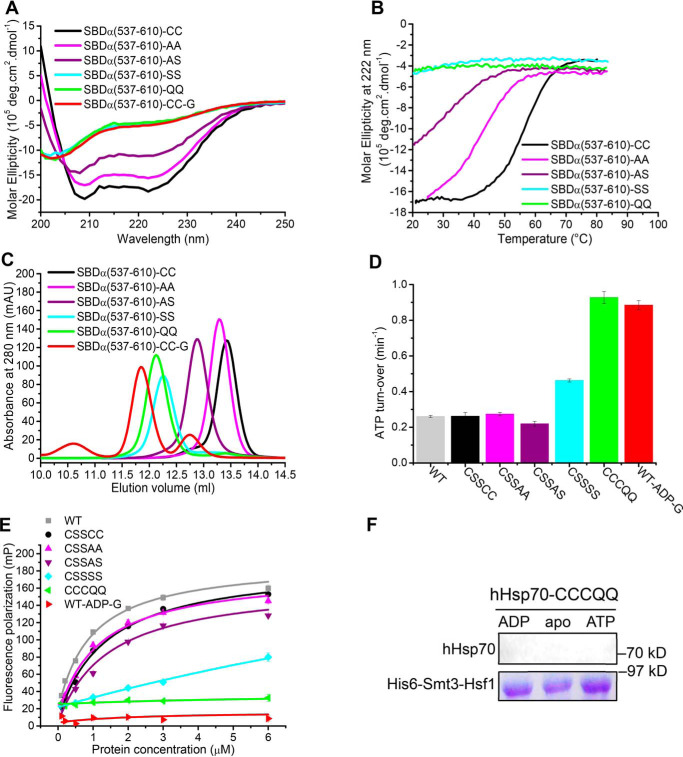
**Mutation or glutathionylation within the SBDα lid of hHsp70 alters the activity of hHsp70 through reducing the stability of the α-helical structure.** *A–C*, conformation and secondary structure of untreated hHsp70 SBDα(537–610) (*-CC*, *black*), SBDα(537–610)-AA (*-AA*, *magenta*), SBDα(537–610)-AS (*-AS*, *purple*), SBDα(537–610)-SS (*-SS*, *cyan*), SBDα(537–610)-QQ (*-QQ*, *green*), and glutathionylated SBDα(537–610) (*-G*, *red*) were compared by far-UV CD (*A*), thermal denaturation monitored by the CD signal at 222 nm (*B*), and SEC (*C*). *D* and *E*, activity of full-length WT and mutant hHsp70 was compared by ATPase activity measured with a malachite green assay (*D*) and peptide binding measured with fluorescence polarization (*E*); untreated WT hHsp70 (*WT-CC*, *gray*), untreated hHsp70-CSSCC (*-CC*, *black*), hHsp70-CSSAA (*-AA*, *magenta*), hHsp70-CSSAS (*-AS*, *purple*), hHsp70-CSSSS (*-SS*, *cyan*), hHsp70-CCCQQ (*-QQ*, *green*), and glutathionylated WT hHsp70 in the presence of 0.5 mm ADP (*WT-ADP-G*, *red*). The data shown are the mean of three independent measurements (each of three replicates), and the *error bars* represent S.E. *F*, interaction of hHsp70-CCCQQ in the absence or in the presence of 1 mm ADP or ATP with Hsf1 was detected by a pulldown assay, performed as in Fig. 6*E*.

Glutathionylation involving Cys-574 and Cys-603 causes disruption of the structure of the SBDα, suggesting that the two Cys residues may be important for the stability of the α-helix bundle. To test this, we mutated each of the two Cys residues to Ala, Ser, and Gln and tested the effect of these mutations on the structure and function of hHsp70. When introduced into the isolated SBDα(537–610), we observed that these mutations affect the structure to different extents in the following order: AA < AS < SS ≤ QQ (Fig. 8, *A–C*). The hydropathy index values of cysteine, alanine, serine, and glutamine are 2.5, 1.8, −0.8, and −3.5, respectively (54), meaning that Ala is most similar to Cys in terms of polarity, although Ser is more similar to Cys in terms of size and shape. Thus, the more significant structural effect of serine mutants than alanine mutants can be attributed to the difference in polarity. Glutamine causes the largest structural changes in the SBDα, consistent with it being the most polar among these residues, together with its relatively bulky side chain. With the increasing polarity of the residues at 574 and 603, a lower α-helical signal and decreased thermal stability was observed by CD, and a larger hydrodynamic volume was observed by SEC (Fig. 8, *A–C*). This indicates that the equilibrium between folding and unfolding shifts toward the unfolded state, and the structure of the molecule is less compact.

The side chains of Cys-574 and Cys-603 are well-buried in the α-helical bundle of the SBDα. They form a hydrophobic core together with the side-chains of Val-552, Val-570, Leu-599, and other hydrophobic residues. Thus, introduction of a polar residue, like serine or glutamine, will disrupt this hydrophobic core and thus disrupt the α-helical bundle. The glutamate residue in GSH has the same hydropathy index value as glutamine (−3.5), and GSH is larger than glutamine. Thus, glutathionylation at Cys-574 and Cys-603 thoroughly disrupts the α-helical bundle structure by introducing a large polar group. As expected, the SBDα(537–610)-QQ mutant shows similarities to the glutathionylated SBDα(537–610) in terms of the far-UV CD spectra and thermal denaturation, although SEC showed a more expanded structure for the glutathionylated protein (Fig. 8, *A–C*). Overall, this indicates that the SBDα(537–610)-QQ mutant is also completely unfolded.

In full-length hHsp70, we observed increased ATPase activity and decreased peptide binding ability of the C-terminal SS and QQ mutants, but no effect of AA and AS mutants (Fig. 8 (*D* and *E*) and Table 4), suggesting that the SBDα region (residues 537–610) in full-length hHsp70 may be more stable than the isolated SBDα(537–610). Comparison of the structures of the isolated SBDα(537–610) and the intact SBD also shows that helices B, C, and D of the isolated SBDα(537–610) form a more compact bundle (Fig. S5); thus, the isolated SBDα(537–610) may have lower tolerance toward mutation. The ATPase activity and peptide binding ability of hHsp70-CCCQQ and glutathionylated ADP-bound hHsp70 were also similar (Fig. 8, *D* and *E*). As expected, hHsp70-CCCQQ cannot bind Hsf1 in the presence or absence of nucleotide, just like glutathionylated hHsp70 (Figs. 6*E* and 8*F*). Thus, C574Q and C603Q mutations can mimic the effects on structure and function of glutathionylation at Cys-574 and Cys-603. From the above results, we conclude that partial or complete unfolding of the SBDα induced by thiol modification or mutation allows this region of sequence to act as pseudo-substrate to bind to the SBDβ, thus promoting ATPase activity and competing with substrate binding.

## Discussion

Although strong oxidative stress is harmful, redox variations are common in cells and facilitate signal transduction for important physiological activities, such as development and differentiation of the embryo (55). Cysteine modifications of proteins provide the main means for redox signal transfer (55). Nearly all known mammalian HspA1A homologues possess five conserved Cys residues, suggesting that these residues may be important for some aspect of Hsp70 function. This study demonstrates that reversible cysteine modification of hHsp70 occurs in cells and investigates the significance of these conserved cysteine residues by testing their reactivity and the potential of glutathionylation to act as a reversible regulatory mechanism. *In vitro* study found that nucleotide binding can protect Cys residues within the NBD of hHsp70 from undergoing oxidative modifications, and in contrast, the relatively high reactivity of Cys residues within the SBD (Cys-574 and Cys-603) is not reduced by nucleotide or substrate binding. Further, it was found that Cys-603 is more reactive and more readily undergoes glutathionylation than Cys-574, but glutathionylation of both Cys-574 and Cys-603 causes more severe structural and functional changes in hHsp70 than glutathionylation of Cys-603 alone. Thus, these two cysteine residues may cooperate to transfer redox signals to Hsp70 client proteins. We found that the glutathionylation/deglutathionylation cycle directs the unfolding and refolding of the SBDα, which blocks or unblocks availability of the substrate-binding site of SBDβ, thus modulating substrate binding, indicating a latent regulatory function of the SBDα distinct from the allosteric regulation induced by nucleotide/substrate binding. It is also suggested that glutathionylation/deglutathionylation could be a switch to turn off/turn on chaperone activity of hHsp70 and thus to transfer redox signals to the clients of hHsp70. This study also found that hHsc70 is not as sensitive to redox as hHsp70, throwing new light on distinct functions of hHsp70 and hHsc70.

### hHsp70 is sensitive to redox changes and is redox-regulated by thiol modification

Most Hsp70s contain at least one Cys residue, but different Hsp70 homologues vary in their distributions of Cys residues. DnaK, which has a single cysteine in the NBD, is found to undergo cysteine modifications, including glutathionylation (26, 37). Glutathionylation also reversibly regulates the structure and function of DnaK under oxidative stress conditions, transferring redox signals to its clients and facilitating initiation of the heat shock response (26). Yeast cytosolic Hsp70 Ssa1, which has three cysteines in the NBD, is also suspected to be a redox sensor (56). hHsc70 (HspA8) has four cysteines, and hHsp70 (HspA1A) has five cysteines. The increasing number of cysteines in Hsp70 with evolution indicates the importance of cysteines for function of Hsp70 and suggests precise division of the roles of different cysteines. It has been reported that the extra Cys-306 in hHsp70 distinguishes redox sensing between hHsp70 and hHsc70 (57). In this study, we observed that the presence of ADP generally protects Cys-306 from undergoing glutathionylation, whereas Cys-603 distinguishes redox sensing between hHsp70 and hHsc70, which is consistent with an earlier study (58). Glutathionylation of hHsc70, but not hHsp70, has been reported previously by a number of different laboratories (21, 22, 33). Because hHsc70 is more abundant than hHsp70, glutathionylation of Hsc70 is more frequently detected both in previous studies and here, although the reactivity/redox sensitivity of hHsp70 is in fact higher (Fig. 7, *A* and *B*). The different reactivity and sensitivity to redox of different cysteines in hHsp70 and among different Hsp70 family members suggests that they have specific physiological functions. Higher redox sensitivity of hHsp70 could be related to its anti-stress function, and lower redox sensitivity of hHsc70 could protect its fundamental cellular function and keep it relatively stable despite variations in the redox environment. This study demonstrates that glutathionylation turns off chaperone activity of hHsp70, which could be a signal for its clients. For example, clients that are protected by or require hHsp70 to facilitate correct folding will show a reduction in activity, whereas clients that are suppressed by hHsp70 will show an increase in activity, leading to varying consequences of redox. Glutathionylation of hHsp70 causes weakening of the interaction between hHsp70 and Hsf1, which could facilitate activation of Hsf1 and initiation of the heat shock response under oxidative stress, similar to the mechanisms observed for *E. coli* DnaK and yeast Ssa1 (26, 56). For the yeast ER Hsp70 homologue Kar2, glutathionylation at Cys-63 in the NBD was also found to serve as a redox sensor, but in this case, the mechanism is different; ATPase activity is abolished, and its affinity for misfolded substrates is enhanced, leading to augmented holdase activity (27, 59). However, whether the mechanism involves activation of Hsf1 (or σ32 in *E. coli*) or enhancement of substrate-binding ability, in each of these cases, glutathionylation of Hsp70 expands the cellular ability to counter the effects of oxidative stress.

In the SBD of DnaK and Ssa1, there are no cysteine residues. In contrast, Cys-574 and Cys-603 in the SBD of hHsp70 can cooperate to sense redox and finely adjust the function of hHsp70, suggesting some specific regulation mechanism involving these two cysteines in mammals. Different cysteine modifications are often detected at a single cysteine residue and in some cases can interconvert (21). Although this study only focuses on glutathionylation, other modifications, such as *S*-nitrosylation of hHsp70 and hHsc70, have been detected by MS (19, 20), showing that cysteine modification of hHsp70 and hHsc70 readily occurs. In addition to the cooperation between Cys-574 and Cys-603 in terms of glutathionylation discussed above, disulfide bond formation between these residues under oxidative conditions is also possible, as predicted in a previous study (58) and detected for SBDα(537–610) by Q-TOF MS detection in this study (Fig. 3*D*). The distance between the thiol groups in Cys-603 and Cys-574 is 4.93 Å according to the crystal structure (PDB code 4PO2, Fig. S5*C*) and 6.69 Å according to the NMR structure (PDB code 2LMG, Fig. S5*C*). The relatively short distance between Cys-603 and Cys-574 suggests a high likelihood of forming a disulfide bond pair between these residues. How it is determined which cysteine modifications will occur at a given cysteine residue of hHsp70, and their different physiological significance, is not yet fully understood.

### Glutathionylation reveals the dynamic nature and regulatory capacity of the SBDα lid

Hsp70 has been shown to be a highly dynamic protein by EPR, NMR, single molecule FRET, and amide hydrogen exchange experiments (13, 28, 60–62). The dynamic nature of the NBD and SBDβ has been well-studied and shown to be the basis of allosteric conformational changes involving the whole Hsp70 molecule (7, 61). However, the dynamics of the hHsp70 SBDα lid (in which the relative positions of helices B, C, and D have been generally assumed to be in a fixed orientation; Fig. S5*A*) are still not fully understood. Amide hydrogen exchange experiments and NMR carried out on the NBD and SBD of DnaK revealed surprisingly high dynamics of α-helix B within the SBDα lid, which facilitates the opening-closing movement of the SBDα lid relative to the SBDβ upon ATP/ADP binding (11, 62). In this study, the observation of the high reactivity of Cys-574 and Cys-603, although they are not surface-exposed in the structure, indicates significant mobility of the individual helices within the α-helix bundle that forms the SBDα lid, allowing molecules such as GSH to reach the buried Cys-574 and Cys-603 residues. Consistent with this, a previous simulation study indicates that the SBDα lid is flexible and can adapt its conformation to facilitate substrate binding (60). In the crystal structure of the hHsp70 SBD, the overall B factor values for the SBDα are higher than those for the SBDβ (8), suggesting higher dynamics of the SBDα lid. The plasticity of the SBDα lid is also indicated by superimposition of the isolated SBDα(537–610) with the intact SBDα/β, which shows that helixes B, C, and D of the isolated SBDα(537–610) form a more compact bundle, and the distance between Cys-574 and Cys-603 is also slightly different in the isolated SBDα(537–610) and intact SBDα/β structures (Fig. S5, *B* and *C*). Thus, the configuration of helices B, C, and D in the SBDα appears to be influenced by the SBDβ.

Based on the dynamic nature of the SBDα lid, it is speculated that its conformation could be influenced by interaction with co-chaperones, binding of different types of substrate, post-translational modifications, and changes in sequence during evolution. Interaction of the SBDα lid with co-chaperones has been demonstrated in an NMR study, and co-chaperones regulate the functional cycle of Hsp70 by altering the conformation of the SBDα lid (48). It is thought that the SBDα lid may provide an interaction site in addition to the SBDβ for large substrates such as aggregates (2). A previous report has shown that methylation at Lys-561 within the SBDα lid decreases interaction of Hsp70 with substrate (17). This study demonstrates that glutathionylation and deglutathionylation at Cys-574 and Cys-603 induces unfolding and refolding of the SBDα lid, which then regulates substrate binding, suggesting a novel Hsp70 regulation mechanism in addition to the well-established allosteric regulation induced by nucleotide/substrate binding. In agreement with our proposal, the SBD structures of DnaK and Hsc70 show that successive deletion of the C-terminal region disrupts the α-helical bundle of the SBDα lid, and the new unfolded region binds to the SBDβ substrate-binding site (50, 52). In the above structures, the same Leu-542 residue in the SBDα lid binds to the hydrophobic site (Fig. 5*D*). Interestingly, the Leu-542 residue in hHsp70 is conserved from *E. coli* to humans (Fig. S6). Due to the intrinsically dynamic nature of the SBDα lid, we would expect that the occurrence of different types of post-translational modification of the Cys, Met, and Tyr residues, due to oxidative stress or other factors, might cause similar unfolding of the SBDα lid as glutathionylation and switch off the chaperone activity of Hsp70 by intramolecular interaction between the conserved Leu and the hydrophobic binding site in the SBDβ. Thus, the proposed switch mechanism may be an important and conserved strategy among members of the Hsp70 family. The sequence of the SBDα lid is the least conserved region of the protein, and Cys-574 and Cys-603 are only conserved in mammalian cytosolic Hsp70. In this study, we found that mutation at Cys-574 and Cys-603 can also alter the stability of the SBDα lid, as well as the intrinsic ATPase activity and substrate binding of the protein, suggesting that the SBDα lid may have contributed to the adaptation of Hsp70 to different cellular environments during evolution.

From the above observations, it can be concluded that factors affecting the stability of the SBDα lid will affect its folding-unfolding equilibrium as well as its interaction with the SBDβ, leading to changes in ATPase activity and substrate binding and, thus, regulation of the functional cycle of Hsp70. This study reveals that the unfolded SBDα lid possesses sufficiently high affinity for the SBDβ that it can act as a pseudo-substrate to compete with external substrates. It was observed for GSK3β that phosphorylation at Ser-9 in the N-terminal flexible region allows this phosphate group to act as a pseudo-substrate, inhibiting secondary phosphorylation of its primary phosphorylated substrate (63). Thus, it could be a common mechanism that post-translational modification helps transform part of a protein into its own ligand, as a means to regulate ligand binding. However, to the best of our knowledge, we present the first such example where post-translational modification induces a significant reversible conformational change, which then causes part of the amino acid chain itself to act as its own substrate.

## Experimental procedures

### Detection of glutathionylation of hHsp70 in HeLa cells by biotin switch assay

#### 

##### Cell culture and treatment

HeLa cells were grown in Dulbecco's modified Eagle's medium (Gibco BRL) supplemented with 10% fetal bovine serum, 100 μg/ml streptomycin, and 100 units/ml penicillin (Gibco BRL) in a humidified 5% CO_2_ incubator at 37 °C. Half of the cultured cells (1.2 × 10^7^, 10 dishes) were treated with 1 or 10 mm diamide for 15 min, the diamide was removed, and then both diamide-treated and untreated cells were incubated with 50 mm NEM for a further 15 min before harvesting. Untreated and diamide-treated cells were each harvested and lysed in 3 ml of ice-cold cell lysis buffer (Beyotime, P0013) with 0.1 m NEM (Sigma–Aldrich) and cOmplete ULTRA protease inhibitor (Roche Applied Science). The soluble part of the cell lysis was separated by centrifugation and subjected to the biotin switch assay.

##### Biotin switch assay

The biotin switch assay was performed following a method applied for cysteine modification detection of yeast ER Hsp70 Kar2 with some modifications (27, 59). The supernatant of the cell lysis (3 ml) was mixed with 12 ml of urea-containing cysteine modification buffer (CMBU; 0.1 m HEPES-NaOH, pH 7.4, 1% SDS, 1 mm EDTA, 8 m urea) with cOmplete ULTRA protease inhibitor (Roche Applied Science) and 0.1 m NEM. Samples were placed for 30 min at room temperature (RT). Proteins were precipitated with 10% TCA on ice for 30 min. The pellet was collected by centrifugation and washed once with 5% TCA and twice with 70% acetone and dissolved in 300 μl of CMBU. Then 6 ml of Grx reduction buffer (0.1 m Tris-HCl, pH 8.0 containing 1 mm EDTA, 0.5 mm GSH, 1 mm NADPH (Sigma–Aldrich), 0.25 units/ml GSH reductase (Sigma–Aldrich) and 60 μg of purified *E. coli* Grx3 C14S/C65Y (27, 33, 38, 44, 64) or Grx3 C11S/C14S/C65Y (27)) was added, and samples were incubated for 15 min at 37 °C. After reduction, samples were quenched with TCA, and protein precipitation was carried out as above. The pellet was dissolved in 300 μl of CMBU with 0.2 mm biotin-maleimide (Sigma–Aldrich) and was placed for 30 min at RT and then protein precipitation was carried out as above. The pellet was dissolved in 100 μl of CMBU.

##### Detection of hHsp70 glutathionylation by Western blotting

Streptavidin beads (Sigma–Aldrich) washed three times with IP buffer (50 mm Tris-HCl, pH 7.4, 0.15 m NaCl, 0.1% SDS) were added into a 40-μl mixture of dissolved proteins in CMBU with 1 ml of IP buffer, and the sample was incubated with rotation at 4 °C overnight. Beads were collected by centrifugation and washed three times with IP buffer, and proteins were eluted for 5 min at 100 °C with 20 μl of 2× SDS-PAGE loading buffer. Proteins were separated by SDS-PAGE, transferred to nitrocellulose, and probed with specific anti-hHsp70 mAb (ABclonal (A1507) or CST (46477)) and anti-GAPDH polyclonal antibody (ABclonal (Ac001) or CST (G9545)) using standard methods. Another 60 μg of total protein of cell lysis was analyzed by Western blotting detection directly with the same antibody as above.

##### Detection of hHsp70 glutathionylation by nanoLC-LTQ-Orbitrap XL MS/MS

The remaining 60 μl of CMBU-dissolved proteins was mixed with 1.5 ml of 50 mm Tris-HCl buffer, pH 8.0. Sequencing grade modified trypsin (Promega) (10 μg) was added, and the digestion system was kept at 37 °C for 24–48 h. After trypsin digestion, 4 m NaCl and 10% SDS were added to adjust the concentration of NaCl and SDS to 0.15 m and 0.1%, respectively. Then streptavidin beads (Sigma–Aldrich) washed three times with IP buffer were added, and the sample was incubated with rotation at 4 °C overnight. Peptide elution and pretreatment before MS measurement were performed as described with slight modifications (65). The beads were collected by centrifugation and subsequently washed with 3 × 100 μl of IP buffer; with 5 × 100 μl of ACN, 1 m NaCl (25:75, v/v); and with 2 × 100 μl with ACN/water (20:80, v/v). Bound peptides were eluted using 20–30 μl of ACN, 10% FA, 2 mm biotin (70:10:20, v/v/v; biotin ∼99%, Sigma–Aldrich) by incubation for 2 h at 37 °C with occasional resuspension. Rotary evaporation of the eluate was performed in a vacuum drier to reduce the volume to 4–6 μl. Then the samples were reconstituted in 0.1% TFA, further desalted using a C18 reverse-phase column (filled with 3-μm ReproSil-Pur C18-AQ from Dr. Maisch GmbH, Ammerbuch), and loaded using a C18 reverse-phase column (filled with 5-μm ReproSil-Pur C18-AQ from Dr. Maisch GmbH, Ammerbuch, Germany) onto LTQ-Orbitrap MS/MS. Data were analyzed using Proteome Discoverer software (version 1.4.0.288; Thermo Fisher Scientific). The second MS spectra were searched in the human database (uniprot_human_proteome_20160229_con) using the SEQUEST search engine. The number of entries in the database actually searched was 92,181. Sequencing grade modified trypsin (Promega) was used to generate peptides. The number of missed and/or nonspecific cleavages permitted was 2. There is no fixed modification, and biotin-maleimide or NEM modification of cysteine and oxidation of methionine were set as variable modifications. The mass tolerance for precursor ions was 20 ppm, and the mass tolerance for fragment ions was 0.5 Da. Peptide-spectrum matching (PSM) was filtered by Percolator calculation, and the false discovery rate was estimated using the *q* value (which was controlled to be <1%). The identified peptides were combined into proteins by the maximum parsimony principle.

The MS proteomics data have been deposited into the ProteomeXchange Consortium via the PRIDE (66) partner repository with the data set identifier PXD017717.

### Protein expression and purification

The human *HSPA1A* gene (67) (UniProtKB code: P0DMV8) and *HSPA8* gene (UniProtKB code: P11142), which were kindly provided by Prof. Richard Morimoto (Northwestern University), were subcloned into the pET28a-smt3 expression plasmid for expression of hHsp70 with a His_6_-Smt3 tag (68). The hHsp70 Cys to Ser or Ala point mutants were created using the Fast site-directed mutagenesis kit (TransGen Biotech). The hHsp70 domain deletion mutants were derived from the human *HSPA1A* gene. The *HSF1* gene, which was cloned from a cDNA library of the HeLa cell line, was subcloned into the pET28a-smt3 expression plasmid for expression with a His_6_-Smt3 tag. The *E. coli* Grx3 expression plasmid was constructed as described (26). The Grx3 C14S/C65Y and Grx3 C11S/C14S/C65Y mutants were created using the Fast site-directed mutagenesis kit (TransGen Biotech). Primer sequences are shown in Table S1. Mutations were confirmed by DNA sequencing.

Expression and purification of hHsp70 and its mutants, hHsc70, Grx3 C14S/C65Y, and Grx3 C11S/C14S/C65Y, were performed as described (26). These proteins were expressed in BL21 (DE3) strain, induced with 0.2 mm isopropyl 1-thio-β-d-galactopyranoside, and grown at 16 °C for 16 h. The harvested cells were lysed using a JNBIO JN-3000 PLUS high-pressure cell press in Buffer A (50 mm Tris-HCl buffer, pH 7.5, 300 mm NaCl) containing 10 mm imidazole and 2 mm β-mercaptoethanol, and the debris was removed by centrifugation (35,000 × *g*, 30 min). The supernatant was then loaded onto a nickel affinity column (chelating Sepharose fast-flow resin; GE Healthcare) and washed with Buffer A containing 40 mm imidazole. Proteins were eluted using Buffer A containing 200 mm imidazole and incubated with Ulp1 at 4 °C for 1 h to remove the His_6_-Smt3 tag. Then the untagged proteins were loaded onto a nickel affinity column again after changing to Buffer A containing 10 mm imidazole, and the run-though was collected for further SEC purification. The concentrated proteins were loaded onto a 120-ml Superdex^TM^ 200 Hiload column (GE Healthcare) equilibrated with Buffer B (50 mm Tris-HCl buffer, pH 7.5, containing 100 mm KCl and 5 mm MgCl_2_). The monomeric peak of hHsp70 was collected for glutathionylation experiments. Hsf1 was purified using the same method as for the other proteins, but excluding the His-smt3 cleavage and the second nickel column purification steps. All protein concentrations are given in terms of monomer and were determined by a bicinchoninic acid (BCA) assay kit (Pierce).

### Measurement of cysteine reactivity of hHsp70 and its mutants

Cysteine reactivity of hHsp70 and its mutants was measured by an Ellman assay as described (69) to predict the possibility of glutathionylation. A standard curve was made using 0–100 μm free cysteine. DTNB (5 μl of 10 mm in 50 mm Na_2_HPO_4_/NaH_2_PO_4_ buffer, pH 7.5) was mixed with hHsp70 or its mutants (145 μl of 10–20 μm), and the absorbance at 412 nm was measured in a Molecular Devices Spectra Max M3 plate reader at RT. If ADP, ATP, or peptide was added, 1 mm ADP/ATP/NRLLLTG peptide was mixed with hHsp70 or its mutants to give a total volume of 145 μl, and the mixture was allowed to stand at RT for 1 h before DTNB was added. The number of active Cys residues can be calculated by dividing the concentration of free thiols in the protein by the concentration of protein.

### Preparation of glutathionylated and deglutathionylated hHsp70 or its mutants

To determine appropriate conditions for glutathionylation, the following conditions were tested: decomposed GSNO (fresh GSNO then placed in the dark at RT for 48 h) of two different concentrations (0.5 mm and 1 mm); fresh GSNO (1 mm) and GSSG (1 mm); GSH (1 mm) and diamide (1 mm); and GSH (1 mm) and H_2_O_2_ (1 or 2 mm). In each case, 15 μm hHsp70 was used, and the sample was placed in the dark at 37 °C for 2 h.

To prepare glutathionylated and deglutathionylated hHsp70, 15 μm hHsp70 (or its mutant) was mixed with 1 mm GSH and 1 mm diamide and allowed to stand in the dark at RT for 1 h to allow glutathionylation. Then 10 mm DTT was added in order to deglutathionylate the protein. GSH, diamide, and DTT were then removed by dialysis. For glutathionylation of ADP-bound full-length hHsp70, ADP (final concentration 1 mm) was added to the protein before GSH and diamide were added.

Western blots were performed as standard to confirm glutathionylation and deglutathionylation of hHsp70 or its mutants. Polyclonal anti-GSH (Millipore, AB5010) at 1:500–1000 dilution was used as the primary antibody for Western blotting detection. Glutathionylation was also confirmed by the absence of free thiols by staining with maleimide-functionalized Alexa Fluor® 350 dye (blue fluorescence). hHsp70 or its mutant was boiled for 10 min to destroy its secondary structure. Cooled protein was mixed with the dye and incubated in the dark at RT for at least 2 h. SDS-PAGE was performed to separate protein and surplus dye. Fluorescence of Alexa Fluor® 350 dye was observed using excitation at 254 nm with a UV lamp. NanoLC-LTQ-Orbitrap XL MS/MS and Q-TOF MS were also performed to detect cysteine modifications of hHsp70 or its mutants.

### Intrinsic fluorescence

Intrinsic fluorescence measurements were carried out on a Hitachi F-4500 or a Shimadzu RF-5301PC instrument. The intrinsic florescence spectra of glutathionylated and deglutathionylated hHsp70 or its mutants were measured between 310 and 400 nm, using excitation wavelengths of 295 nm at 25 °C. The proteins were prepared in Buffer B.

To monitor the shift of CSM for the intrinsic fluorescence spectra caused by glutathionylation of hHsp70, 1 mm diamide with 1 mm GSH, 4 mm H_2_O_2_ with 1 mm GSH, or 2 mm GSSG was rapidly mixed with 10 μm WT hHsp70 in Buffer B in the presence of 0.5 mm ADP before the spectra were recorded at 37 °C every 30 s (for fast reactions) or 10 min (for slow reactions) until the intrinsic fluorescence signal reached a plateau; spectra were recorded between 310 and 380 nm with excitation at 295 nm. When monitoring the CSM shift caused by deglutathionylation of hHsp70, 10 mm DTT was added and mixed rapidly before spectrum acquisition. The CSM of intrinsic fluorescence was calculated using the following formula.

(Eq. 1) $$CSM=\frac{\sum_{i=310}^{380}i\times IF_{i}}{\sum_{i=310}^{380}IF_{i}}$$

### CD

Far-UV CD spectra were measured between 200 and 250 nm on a Chirascan Plus CD instrument (Applied Photophysics) at 25 °C in a 1-mm path length thermostatted cuvette after preincubation for 10 min at 25 °C. Spectra of glutathionylated and deglutathionylated hHsp70 or its mutants were compared in Buffer B.

Temperature-induced denaturation measurements were performed under the following conditions. 5 μm hHsp70 truncation mutants were prepared in Buffer B. Denaturation was followed by monitoring of the increase in ellipticity at 222 nm. A temperature ramp of 0.5 °C/min was applied between 25 and 95 °C. All equilibrium measurements were performed using a Chirascan Plus CD instrument (Applied Photophysics) in a 1-mm path-length thermostatted quartz cuvette. Data were collected with a band pass of 1 nm, and the sensitivity was set to 100 millidegrees.

### Size-exclusion chromatography assay

The oligomeric states of glutathionylated and deglutathionylated hHsp70 or its mutants were compared by size-exclusion chromatography (Superdex 200 10/300 GL column or Superdex 75 10/300 GL column, GE Healthcare) in Buffer B at RT. β-Amylase (200 kDa), alcohol dehydrogenase (150 kDa), BSA (66 kDa), ovalbumin (45 kDa), carbonic anhydrase (29 kDa), phenylmethylsulfonyl fluoride-treated trypsinogen (24 kDa), and cytochrome *c* (12.4 kDa) were used as molecular mass standards.

### NMR experiments and structure calculations

^15^N-Labeled hHsp70(537–610) and hHsp70(385–641) were prepared using the same procedures as for WT hHsp70, except that cells were grown in M9 minimal medium containing ^15^NH_4_Cl as the sole nitrogen source. NMR experiments were performed at RT on an Agilent DD2 (DirectDrive 2) 600-MHz spectrometer equipped with a cryoprobe. The ^1^H-^15^N HSQC spectra of 0.2 mm hHsp70(537–610) and hHsp70(385–641) were acquired in Buffer B with 10% (v/v) D_2_O for nonglutathionylated (*i.e.* control), glutathionylated, and deglutathionylated samples. For the nonglutathionylated sample, a final concentration of 2 mm GSH was added to the ^15^N-labeled hHsp70(537–610) or hHsp70(385–641). Fresh diamide (2 mm) was added to promote glutathionylation of hHsp70(537–610) and hHsp70(385–641) to obtain glutathionylated samples. Then 10 mm DTT was added to reduce them to obtain deglutathionylated samples.

^15^N-^13^C–labeled hHsp70(385–641) was prepared using the same procedures as for WT hHsp70, except that cells were grown in M9 minimal medium containing ^15^NH_4_Cl and [^13^C]glucose as the sole nitrogen and carbon sources. NMR experiments were performed at 308 K on Bruker AVANCE 600- and 950-MHz spectrometers equipped with cryoprobes. ^15^N-^13^C–labeled glutathionylated hHsp70(385–641) was prepared using the same procedures as WT hHsp70 and was concentrated to 0.5 mm. NMR samples of glutathionylated hHsp70(385–641) contained 0.5 mm protein in 10 mm sodium phosphate buffer, pH 7.0, 5 mm DTT, 2 mm EDTA, 0.02% (w/v) sodium 2,2-dimethylsilapentane-5-sulfonate, and 10% (v/v) D_2_O. Two-dimensional ^1^H-^15^N and ^1^H-^13^C HSQC and three-dimensional CBCA(CO)NH, HNCACB, HNCO, HN(CA)CO, HNCA, HN(CO)CA, HBHA(CO)NH, HBHANH, HCCH-TOCSY, and CCH-TOCSY experiments were performed to obtain backbone and side-chain assignments of glutathionylated hHsp70(385–641). Three-dimensional ^1^H-^15^N, ^1^H-^13^C, and ^1^H-^13^C NOESY-HSQC spectra were collected to generate distance restraints. All data were processed with NMRPipe (70) and analyzed with NMRViewJ (71). Proton chemical shifts were referenced to the internal sodium 2,2-dimethylsilapentane-5-sulfonate, and ^15^N and ^13^C chemical shifts were referenced indirectly (72).

The structures of glutathionylated hHsp70(385–641) were initially calculated with the program CYANA (73) and then refined using CNS (74) with manual assignments as well as semiautomated NOE assignments by SANE (75). Backbone dihedral angle restraints obtained using CSI (76) and TalosN (77) as well as hydrogen bond restraints according to the regular secondary structure patterns were also incorporated into the structural calculation. From 100 CNS-calculated structures, the 50 lowest-energy conformers of the glutathionylated hHsp70(385–641) were selected for further water refinement using CNS and RECOORDScript (78). The resulting 20 energy-minimized conformers were used to represent the solution structure of glutathionylated hHsp70(385–641). The quality of the determined structures (Table 3) was analyzed using PROCHECK-NMR (79) and MolMol (80). Structural figures were created with MolMol (80) and PyMOL (81).

### ATPase assay (malachite green)

Colorimetric determination of P_i_ produced by ATP hydrolysis was performed using the malachite green reagent, prepared as described (82, 83). A 10-μl volume of glutathionylated/deglutathionylated hHsp70 or its mutants (1 μm) was mixed with 10 μl of 2 mm ATP in Buffer B in a 96-well plate. The plate was incubated for 4 h at 37 °C. An 80-μl volume of malachite green and 10 μl of 34% sodium citrate were added sequentially. The samples were mixed thoroughly and incubated at 37 °C for 30 min before measuring the *A*_620_ on a SpectraMax M3e plate reader (Molecular Devices). The rate of intrinsic ATP hydrolysis was deduced by subtracting the signal from ATP in the absence of chaperone.

### Peptide-binding assay

Peptide-binding assays based on fluorescence polarization were performed as described previously with slight modifications (84). Steady-state FP measurements were performed at RT with a 60-min incubation in Buffer B to give the dissociation constant (*K_d_*). Binding was assessed by incubating increasing concentrations of control, glutathionylated, or deglutathionylated hHsp70 or its mutants with a fixed concentration (20 nm) of fluorescently labeled substrate (FITC-ALLLSAPRR peptide, FAR), and FP values were measured. FP measurements were performed on a Fluostar microplate reader (BMG Labtech) using the FP filter set (emission 485 nm and excitation 520 nm). FP values are expressed in millipolarization units (mP). All statistical analyses were performed with Origin software. Binding data were analyzed using nonlinear regression analysis (single site binding model) in Origin software. Kinetic FP measurements were performed by monitoring the time course of peptide binding at RT. After rapid mixing of 20 nm FAR and 10 μm hHsp70 or its mutants in the absence or in the presence of 1 mm ADP/ATP, FP was recorded against time. Peptide-bound hHsp70 (or its mutants) in the absence or in the presence of nucleotide was glutathionylated (or oxidized) by the addition of 1 mm diamide with 1 mm GSH (or 1 mm diamide alone) at the 60-min time point and then deglutathionylated (reduced) by the addition of 10 mm DTT at the 120-min time point.

### Pulldown assay

To determine interactions between Hsf1 and control, glutathionylated, or deglutathionylated hHsp70 or its mutants, pulldown assays were performed as described previously with slight modifications (85). In brief, 2 μm His_6_-Smt3-Hsf1 and 20 μm hHsp70 or its mutants were incubated in Buffer B in the presence of 1 mm ADP or ATP at 8 °C for 60 min. The protein complex was precipitated with nickel-Sepharose HP (GE Healthcare). After washing the nickel-Sepharose resin with Buffer C (50 mm Tris-HCl, pH 7.5, 300 mm KCl, 5 mm MgCl_2_) containing 40 mm imdazole, the bound protein was eluted with Buffer C containing 300 mm imidazole. The eluted proteins were analyzed by SDS-PAGE followed by staining with Coomassie Brilliant Blue to check for the presence of an interaction.

## Data availability

The atomic coordinates and structure factors have been deposited in the Protein Data Bank under accession code 5GJJ. The MS proteomics data have been deposited into the ProteomeXchange Consortium via the PRIDE partner repository with the data set identifier PXD017717. All other data needed to evaluate the conclusions are present in the article and/or the supporting information.

## Supplementary Material

Supporting Information

## References

[1] Kim Y. E., Hipp M. S., Bracher A., Hayer-Hartl M., and Hartl F. U. (2013) Molecular chaperone functions in protein folding and proteostasis. Annu. Rev. Biochem. 82, 323–355 10.1146/annurev-biochem-060208-092442 23746257

[2] Clerico E. M., Tilitsky J. M., Meng W., and Gierasch L. M. (2015) How hsp70 molecular machines interact with their substrates to mediate diverse physiological functions. J. Mol. Biol. 427, 1575–1588 10.1016/j.jmb.2015.02.004 25683596 PMC4440321

[3] Evans C. G., Chang L., and Gestwicki J. E. (2010) Heat shock protein 70 (hsp70) as an emerging drug target. J. Med. Chem. 53, 4585–4602 10.1021/jm100054f 20334364 PMC2895966

[4] Zylicz M., and Wawrzynow A. (2001) Insights into the function of Hsp70 chaperones. IUBMB Life 51, 283–287 10.1080/152165401317190770 11699872

[5] Schlecht R., Scholz S. R., Dahmen H., Wegener A., Sirrenberg C., Musil D., Bomke J., Eggenweiler H. M., Mayer M. P., and Bukau B. (2013) Functional analysis of Hsp70 inhibitors. PLoS ONE 8, e78443 10.1371/journal.pone.0078443 24265689 PMC3827032

[6] Bertelsen E. B., Chang L., Gestwicki J. E., and Zuiderweg E. R. (2009) Solution conformation of wild-type *E. coli* Hsp70 (DnaK) chaperone complexed with ADP and substrate. Proc. Natl. Acad. Sci. U.S.A. 106, 8471–8476 10.1073/pnas.0903503106 19439666 PMC2689011

[7] Zhuravleva A., and Gierasch L. M. (2011) Allosteric signal transmission in the nucleotide-binding domain of 70-kDa heat shock protein (Hsp70) molecular chaperones. Proc. Natl. Acad. Sci. U.S.A. 108, 6987–6992 10.1073/pnas.1014448108 21482798 PMC3084084

[8] Zhang P., Leu J. I., Murphy M. E., George D. L., and Marmorstein R. (2014) Crystal structure of the stress-inducible human heat shock protein 70 substrate-binding domain in complex with peptide substrate. PLoS ONE 9, e103518 10.1371/journal.pone.0103518 25058147 PMC4110032

[9] Zuiderweg E. R., Bertelsen E. B., Rousaki A., Mayer M. P., Gestwicki J. E., and Ahmad A. (2013) Allostery in the Hsp70 chaperone proteins. Top. Curr. Chem. 328, 99–153 10.1007/128_2012_323 22576356 PMC3623542

[10] Vogel M., Mayer M. P., and Bukau B. (2006) Allosteric regulation of Hsp70 chaperones involves a conserved interdomain linker. J. Biol. Chem. 281, 38705–38711 10.1074/jbc.M609020200 17052976

[11] Zhuravleva A., Clerico E. M., and Gierasch L. M. (2012) An interdomain energetic tug-of-war creates the allosterically active state in Hsp70 molecular chaperones. Cell 151, 1296–1307 10.1016/j.cell.2012.11.002 23217711 PMC3521165

[12] Qi R., Sarbeng E. B., Liu Q., Le K. Q., Xu X., Xu H., Yang J., Wong J. L., Vorvis C., Hendrickson W. A., Zhou L., and Liu Q. (2013) Allosteric opening of the polypeptide-binding site when an Hsp70 binds ATP. Nat. Struct. Mol. Biol. 20, 900–907 10.1038/nsmb.2583 23708608 PMC3772632

[13] Kityk R., Kopp J., Sinning I., and Mayer M. P. (2012) Structure and dynamics of the ATP-bound open conformation of Hsp70 chaperones. Mol. Cell 48, 863–874 10.1016/j.molcel.2012.09.023 23123194

[14] Truman A. W., Kristjansdottir K., Wolfgeher D., Hasin N., Polier S., Zhang H., Perrett S., Prodromou C., Jones G. W., and Kron S. J. (2012) CDK-dependent Hsp70 Phosphorylation controls G_1_ cyclin abundance and cell-cycle progression. Cell 151, 1308–1318 10.1016/j.cell.2012.10.051 23217712 PMC3778871

[15] Choudhary C., Kumar C., Gnad F., Nielsen M. L., Rehman M., Walther T. C., Olsen J. V., and Mann M. (2009) Lysine acetylation targets protein complexes and co-regulates major cellular functions. Science 325, 834–840 10.1126/science.1175371 19608861

[16] Soss S. E., Rose K. L., Hill S., Jouan S., and Chazin W. J. (2015) Biochemical and proteomic analysis of ubiquitination of Hsc70 and Hsp70 by the E3 ligase CHIP. PLoS ONE 10, e0128240 10.1371/journal.pone.0128240 26010904 PMC4444009

[17] Jakobsson M. E., Moen A., Bousset L., Egge-Jacobsen W., Kernstock S., Melki R., and Falnes P. Ø. (2013) Identification and characterization of a novel human methyltransferase modulating Hsp70 protein function through lysine methylation. J. Biol. Chem. 288, 27752–27763 10.1074/jbc.M113.483248 23921388 PMC3784692

[18] Je J. H., Kim D. Y., Roh H. J., Pak C., Kim D. H., Byamba D., Jee H., Kim T. G., Park J. M., Lee S. K., and Lee M. G. (2013) The antioxidative effect of heat-shock protein 70 in dendritic cells. Scand. J. Immunol. 78, 238–247 10.1111/sji.12078 23679814

[19] Huang B., Chen S. C., and Wang D. L. (2009) Shear flow increases *S*-nitrosylation of proteins in endothelial cells. Cardiovasc. Res. 83, 536–546 10.1093/cvr/cvp154 19447776

[20] Lefièvre L., Chen Y., Conner S. J., Scott J. L., Publicover S. J., Ford W. C., and Barratt C. L. (2007) Human spermatozoa contain multiple targets for protein *S*-nitrosylation: an alternative mechanism of the modulation of sperm function by nitric oxide? Proteomics 7, 3066–3084 10.1002/pmic.200700254 17683036 PMC2777308

[21] Fratelli M., Gianazza E., and Ghezzi P. (2004) Redox proteomics: identification and functional role of glutathionylated proteins. Expert Rev. Proteomics 1, 365–376 10.1586/14789450.1.3.365 15966832

[22] Hoppe G., Chai Y. C., Crabb J. W., and Sears J. (2004) Protein *S*-glutathionylation in retinal pigment epithelium converts heat shock protein 70 to an active chaperone. Exp. Eye Res. 78, 1085–1092 10.1016/j.exer.2004.02.001 15109915

[23] Michelet L., Zaffagnini M., Vanacker H., Le Maréchal P., Marchand C., Schroda M., Lemaire S. D., and Decottignies P. (2008) *In vivo* targets of *S*-thiolation in *Chlamydomonas reinhardtii*. J. Biol. Chem. 283, 21571–21578 10.1074/jbc.M802331200 18534986

[24] Konstantinidis D., Paletas K., Koliakos G., and Kaloyianni M. (2009) The ambiguous role of the Na^+^-H^+^ exchanger isoform 1 (NHE1) in leptin-induced oxidative stress in human monocytes. Cell Stress Chaperones 14, 591–601 10.1007/s12192-009-0110-4 19301149 PMC2866947

[25] Ansong C., Wu S., Meng D., Liu X., Brewer H. M., Deatherage Kaiser B. L., Nakayasu E. S., Cort J. R., Pevzner P., Smith R. D., Heffron F., Adkins J. N., and Pasa-Tolic L. (2013) Top-down proteomics reveals a unique protein *S*-thiolation switch in *Salmonella Typhimurium* in response to infection-like conditions. Proc. Natl. Acad. Sci. U.S.A. 110, 10153–10158 10.1073/pnas.1221210110 23720318 PMC3690903

[26] Zhang H., Yang J., Wu S., Gong W., Chen C., and Perrett S. (2016) Glutathionylation of the bacterial Hsp70 chaperone DnaK provides a link between oxidative stress and the heat shock response. J. Biol. Chem. 291, 6967–6981 10.1074/jbc.M115.673608 26823468 PMC4807281

[27] Wang J., and Sevier C. S. (2016) Formation and reversibility of BiP protein cysteine oxidation facilitate cell survival during and post oxidative stress. J. Biol. Chem. 291, 7541–7557 10.1074/jbc.M115.694810 26865632 PMC4817183

[28] Mayer M. P. (2013) Hsp70 chaperone dynamics and molecular mechanism. Trends Biochem. Sci. 38, 507–514 10.1016/j.tibs.2013.08.001 24012426

[29] Yang J., Carroll K. S., and Liebler D. C. (2016) The expanding landscape of the thiol redox proteome. Mol. Cell. Proteomics 15, 1–11 10.1074/mcp.O115.056051 26518762 PMC4762510

[30] Dalle-Donne I., Rossi R., Colombo G., Giustarini D., and Milzani A. (2009) Protein *S*-glutathionylation: a regulatory device from bacteria to humans. Trends Biochem. Sci. 34, 85–96 10.1016/j.tibs.2008.11.002 19135374

[31] Filomeni G., Rotilio G., and Ciriolo M. R. (2005) Disulfide relays and phosphorylative cascades: partners in redox-mediated signaling pathways. Cell Death Differ. 12, 1555–1563 10.1038/sj.cdd.4401754 16151458

[32] Zhang J., Ye Z. W., Singh S., Townsend D. M., and Tew K. D. (2018) An evolving understanding of the *S*-glutathionylation cycle in pathways of redox regulation. Free Radic. Biol. Med. 120, 204–216 10.1016/j.freeradbiomed.2018.03.038 29578070 PMC5940525

[33] Su D., Gaffrey M. J., Guo J., Hatchell K. E., Chu R. K., Clauss T. R., Aldrich J. T., Wu S., Purvine S., Camp D. G., Smith R. D., Thrall B. D., and Qian W. J. (2014) Proteomic identification and quantification of *S*-glutathionylation in mouse macrophages using resin-assisted enrichment and isobaric labeling. Free Radic. Biol. Med. 67, 460–470 10.1016/j.freeradbiomed.2013.12.004 24333276 PMC3945121

[34] Pastore A., and Piemonte F. (2012) *S*-Glutathionylation signaling in cell biology: progress and prospects. Eur. J. Pharm. Sci. 46, 279–292 10.1016/j.ejps.2012.03.010 22484331

[35] Mieyal J. J., and Chock P. B. (2012) Posttranslational modification of cysteine in redox signaling and oxidative stress: Focus on *S*-glutathionylation. Antioxid. Redox Signal. 16, 471–475 10.1089/ars.2011.4454 22136616 PMC3270050

[36] Penna C., Sorge M., Femminò S., Pagliaro P., and Brancaccio M. (2018) Redox aspects of chaperones in cardiac function. Front. Physiol. 9, 216 10.3389/fphys.2018.00216 29615920 PMC5864891

[37] Winter J., Linke K., Jatzek A., and Jakob U. (2005) Severe oxidative stress causes inactivation of DnaK and activation of the redox-regulated chaperone Hsp33. Mol. Cell 17, 381–392 10.1016/j.molcel.2004.12.027 15694339

[38] Duan J., Kodali V. K., Gaffrey M. J., Guo J., Chu R. K., Camp D. G., Smith R. D., Thrall B. D., and Qian W. J. (2016) Quantitative profiling of protein *S*-glutathionylation reveals redox-dependent regulation of macrophage function during nanoparticle-induced oxidative stress. ACS Nano 10, 524–538 10.1021/acsnano.5b05524 26700264 PMC4762218

[39] Guo J., Gaffrey M. J., Su D., Liu T., Camp D. G. 2nd, Smith R. D., and Qian W. J. (2014) Resin-assisted enrichment of thiols as a general strategy for proteomic profiling of cysteine-based reversible modifications. Nat. Protoc. 9, 64–75 10.1038/nprot.2013.161 24336471 PMC4038159

[40] Li R., and Kast J. (2017) Biotin switch assays for quantitation of reversible cysteine oxidation. Methods Enzymol. 585, 269–284 10.1016/bs.mie.2016.10.006 28109433

[41] Li R., Huang J., and Kast J. (2015) Identification of total reversible cysteine oxidation in an atherosclerosis model using a modified biotin switch assay. J. Proteome Res. 14, 2026–2035 10.1021/acs.jproteome.5b00133 25767911

[42] Mohr S., Hallak H., de Boitte A., Lapetina E. G., and Brüne B. (1999) Nitric oxide-induced *S*-glutathionylation and inactivation of glyceraldehyde-3-phosphate dehydrogenase. J. Biol. Chem. 274, 9427–9430 10.1074/jbc.274.14.9427 10092623

[43] Yap L. P., Garcia J. V., Han D. S., and Cadenas E. (2010) Role of nitric oxide-mediated glutathionylation in neuronal function: potential regulation of energy utilization. Biochem. J. 428, 85–93 10.1042/BJ20100164 20210787 PMC3042800

[44] Nordstrand K., Åslund F., Holmgren A., Otting G., and Berndt K. D. (1999) NMR structure of *Escherichia coli* glutaredoxin 3-glutathione mixed disulfide complex: implications for the enzymatic mechanism. J. Mol. Biol. 286, 541–552 10.1006/jmbi.1998.2444 9973569

[45] Kosower N. S., and Kosower E. M. (1995) Diamide: an oxidant probe for thiols. Methods Enzymol. 251, 123–133 10.1016/0076-6879(95)51116-4 7651192

[46] McCarty J. S., and Walker G. C. (1991) DnaK as a thermometer: threonine-199 is site of autophosphorylation and is critical for ATPase activity. Proc. Natl. Acad. Sci. U.S.A. 88, 9513–9517 10.1073/pnas.88.21.9513 1835085 PMC52748

[47] Tao L., and English A. M. (2004) Protein *S*-glutathiolation triggered by decomposed *S*-nitrosoglutathione. Biochemistry 43, 4028–4038 10.1021/bi035924o 15049710

[48] Gao X. C., Zhou C. J., Zhou Z. R., Wu M., Cao C. Y., and Hu H. Y. (2012) The C-terminal helices of heat shock protein 70 are essential for J-domain binding and ATPase activation. J. Biol. Chem. 287, 6044–6052 10.1074/jbc.M111.294728 22219199 PMC3285371

[49] Hassan A. Q., Kirby C. A., Zhou W., Schuhmann T., Kityk R., Kipp D. R., Baird J., Chen J., Chen Y., Chung F., Hoepfner D., Movva N. R., Pagliarini R., Petersen F., Quinn C., et al (2015) The novolactone natural product disrupts the allosteric regulation of Hsp70. Chem. Biol. 22, 87–97 10.1016/j.chembiol.2014.11.007 25544045

[50] Morshauser R. C., Hu W., Wang H., Pang Y., Flynn G. C., and Zuiderweg E. R. (1999) High-resolution solution structure of the 18 kDa substrate-binding domain of the mammalian chaperone protein Hsc70. J. Mol. Biol. 289, 1387–1403 10.1006/jmbi.1999.2776 10373374

[51] Jiang J., Prasad K., Lafer E. M., and Sousa R. (2005) Structural basis of interdomain communication in the Hsc70 chaperone. Mol. Cell 20, 513–524 10.1016/j.molcel.2005.09.028 16307916 PMC4443753

[52] Wang H., Kurochkin A. V., Pang Y., Hu W., Flynn G. C., and Zuiderweg E. R. (1998) NMR solution structure of the 21 kDa chaperone protein DnaK substrate binding domain: a preview of chaperone-protein interaction. Biochemistry 37, 7929–7940 10.1021/bi9800855 9609686

[53] Abravaya K., Myers M. P., Murphy S. P., and Morimoto R. I. (1992) The human heat shock protein hsp70 interacts with HSF, the transcription factor that regulates heat shock gene expression. Genes Dev. 6, 1153–1164 10.1101/gad.6.7.1153 1628823

[54] Kyte J., and Doolittle R. F. (1982) A simple method for displaying the hydropathic character of a protein. J. Mol. Biol. 157, 105–132 10.1016/0022-2836(82)90515-0 7108955

[55] Wani R., and Murray B. W. (2017) Analysis of cysteine redox post-translational modifications in cell biology and drug pharmacology. Methods Mol. Biol. 1558, 191–212 10.1007/978-1-4939-6783-4_9 28150239

[56] Wang Y., Gibney P. A., West J. D., and Morano K. A. (2012) The yeast Hsp70 Ssa1 is a sensor for activation of the heat shock response by thiol-reactive compounds. Mol. Biol. Cell 23, 3290–3298 10.1091/mbc.e12-06-0447 22809627 PMC3469052

[57] Miyata Y., Rauch J. N., Jinwal U. K., Thompson A. D., Srinivasan S., Dickey C. A., and Gestwicki J. E. (2012) Cysteine reactivity distinguishes redox sensing by the heat-inducible and constitutive forms of heat shock protein 70. Chem. Biol. 19, 1391–1399 10.1016/j.chembiol.2012.07.026 23177194 PMC3508472

[58] Callahan M. K., Chaillot D., Jacquin C., Clark P. R., and Ménoret A. (2002) Differential acquisition of antigenic peptides by Hsp70 and Hsc70 under oxidative conditions. J. Biol. Chem. 277, 33604–33609 10.1074/jbc.M202890200 12114509

[59] Wang J., Pareja K. A., Kaiser C. A., and Sevier C. S. (2014) Redox signaling via the molecular chaperone BiP protects cells against endoplasmic reticulum-derived oxidative stress. Elife 3, e03496 10.7554/eLife.03496 25053742 PMC4132286

[60] Azoulay I., Kucherenko N., Nachliel E., Gutman M., Azem A., and Tsfadia Y. (2013) Tracking the interplay between bound peptide and the lid domain of DnaK, using molecular dynamics. Int. J. Mol. Sci. 14, 12675–12695 10.3390/ijms140612675 23774839 PMC3709807

[61] Zhuravleva A., and Gierasch L. M. (2015) Substrate-binding domain conformational dynamics mediate Hsp70 allostery. Proc. Natl. Acad. Sci. U.S.A. 112, E2865–E2873 10.1073/pnas.1506692112 26038563 PMC4460500

[62] Rist W., Graf C., Bukau B., and Mayer M. P. (2006) Amide hydrogen exchange reveals conformational changes in hsp70 chaperones important for allosteric regulation. J. Biol. Chem. 281, 16493–16501 10.1074/jbc.M600847200 16613854

[63] Beurel E., Grieco S. F., and Jope R. S. (2015) Glycogen synthase kinase-3 (GSK3): regulation, actions, and diseases. Pharmacol. Ther. 148, 114–131 10.1016/j.pharmthera.2014.11.016 25435019 PMC4340754

[64] Lind C., Gerdes R., Hamnell Y., Schuppe-Koistinen I., von Löwenhielm H. B., Holmgren A., and Cotgreave I. A. (2002) Identification of *S*-glutathionylated cellular proteins during oxidative stress and constitutive metabolism by affinity purification and proteomic analysis. Arch. Biochem. Biophys. 406, 229–240 10.1016/S0003-9861(02)00468-X 12361711

[65] Tutturen A. E., Holm A., and Fleckenstein B. (2013) Specific biotinylation and sensitive enrichment of citrullinated peptides. Anal. Bioanal. Chem. 405, 9321–9331 10.1007/s00216-013-7376-1 24081567

[66] Perez-Riverol Y., Csordas A., Bai J., Bernal-Llinares M., Hewapathirana S., Kundu D. J., Inuganti A., Griss J., Mayer G., Eisenacher M., Pérez E., Uszkoreit J., Pfeuffer J., Sachsenberg T., Yilmaz S., et al (2019) The PRIDE database and related tools and resources in 2019: improving support for quantification data. Nucleic Acids Res. 47, D442–D450 10.1093/nar/gky1106 30395289 PMC6323896

[67] Hunt C., and Morimoto R. I. (1985) Conserved features of eukaryotic hsp70 genes revealed by comparison with the nucleotide sequence of human hsp70. Proc. Natl. Acad. Sci. U.S.A. 82, 6455–6459 10.1073/pnas.82.19.6455 3931075 PMC390735

[68] Mossessova E., and Lima C. D. (2000) Ulp1-SUMO crystal structure and genetic analysis reveal conserved interactions and a regulatory element essential for cell growth in yeast. Mol. Cell 5, 865–876 10.1016/S1097-2765(00)80326-3 10882122

[69] Riddles P. W., Blakeley R. L., and Zerner B. (1979) Ellman's reagent: 5,5′-dithiobis(2-nitrobenzoic acid)—a reexamination. Anal. Biochem. 94, 75–81 10.1016/0003-2697(79)90792-9 37780

[70] Delaglio F., Grzesiek S., Vuister G. W., Zhu G., Pfeifer J., and Bax A. (1995) NMRPipe: a multidimensional spectral processing system based on UNIX pipes. J. Biomol. NMR 6, 277–293 10.1007/bf00197809 8520220

[71] Johnson B. A., and Blevins R. A. (1994) NMR View: A computer program for the visualization and analysis of NMR data. J. Biomol. NMR 4, 603–614 10.1007/BF00404272 22911360

[72] Markley J. L., Bax A., Arata Y., Hilbers C. W., Kaptein R., Sykes B. D., Wright P. E., and Wüthrich K. (1998) Recommendations for the presentation of NMR structures of proteins and nucleic acids. J. Mol. Biol. 280, 933–952 10.1006/jmbi.1998.1852 9671561

[73] Herrmann T., Güntert P., and Wüthrich K. (2002) Protein NMR structure determination with automated NOE assignment using the new software CANDID and the torsion angle dynamics algorithm DYANA. J. Mol. Biol. 319, 209–227 10.1016/S0022-2836(02)00241-3 12051947

[74] Brünger A. T., Adams P. D., Clore G. M., DeLano W. L., Gros P., Grosse-Kunstleve R. W., Jiang J. S., Kuszewski J., Nilges M., Pannu N. S., Read R. J., Rice L. M., Simonson T., and Warren G. L. (1998) Crystallography & NMR system: A new software suite for macromolecular structure determination. Acta Crystallogr. D Biol. Crystallogr. 54, 905–921 10.1107/S0907444998003254 9757107

[75] Duggan B. M., Legge G. B., Dyson H. J., and Wright P. E. (2001) SANE (Structure Assisted NOE Evaluation): an automated model-based approach for NOE assignment. J. Biomol. NMR 19, 321–329 10.1023/A:1011227824104 11370778

[76] Wishart D. S., and Sykes B. D. (1994) The ^13^C chemical-shift index: a simple method for the identification of protein secondary structure using ^13^C chemical-shift data. J. Biomol. NMR 4, 171–180 10.1007/bf00175245 8019132

[77] Shen Y., and Bax A. (2013) Protein backbone and sidechain torsion angles predicted from NMR chemical shifts using artificial neural networks. J. Biomol. NMR 56, 227–241 10.1007/s10858-013-9741-y 23728592 PMC3701756

[78] Nederveen A. J., Doreleijers J. F., Vranken W., Miller Z., Spronk C. A., Nabuurs S. B., Güntert P., Livny M., Markley J. L., Nilges M., Ulrich E. L., Kaptein R., and Bonvin A. M. (2005) RECOORD: a recalculated coordinate database of 500+ proteins from the PDB using restraints from the BioMagResBank. Proteins 59, 662–672 10.1002/prot.20408 15822098

[79] Laskowski R. A., Rullmannn J. A., MacArthur M. W., Kaptein R., and Thornton J. M. (1996) AQUA and PROCHECK-NMR: programs for checking the quality of protein structures solved by NMR. J. Biomol. NMR 8, 477–486 10.1007/bf00228148 9008363

[80] Koradi R., Billeter M., and Wüthrich K. (1996) MOLMOL: a program for display and analysis of macromolecular structures. J. Mol. Graph. 14, 51–55, 29–32 10.1016/0263-7855(96)00009-4 8744573

[81] DeLano W. L. (2012) The PyMOL Molecular Graphics System, version 1.5.0.1, Schroedinger, LLC, New York

[82] Chang L., Bertelsen E. B., Wisén S., Larsen E. M., Zuiderweg E. R., and Gestwicki J. E. (2008) High-throughput screen for small molecules that modulate the ATPase activity of the molecular chaperone DnaK. Anal. Biochem. 372, 167–176 10.1016/j.ab.2007.08.020 17904512

[83] Zhang H., Loovers H. M., Xu L. Q., Wang M., Rowling P. J., Itzhaki L. S., Gong W., Zhou J. M., Jones G. W., and Perrett S. (2009) Alcohol oxidase (AOX1) from *Pichia pastoris* is a novel inhibitor of prion propagation and a potential ATPase. Mol. Microbiol. 71, 702–716 10.1111/j.1365-2958.2008.06557.x 19040632

[84] Ricci L., and Williams K. P. (2008) Development of fluorescence polarization assays for the molecular chaperone Hsp70 family members: Hsp72 and DnaK. Curr. Chem. Genomics 2, 90–95 10.2174/1875397300802010090 20161846 PMC2803438

[85] Noguchi A., Ikeda A., Mezaki M., Fukumori Y., and Kanemori M. (2014) DnaJ-promoted binding of DnaK to multiple sites on σ32 in the presence of ATP. J. Bacteriol. 196, 1694–1703 10.1128/JB.01197-13 24532774 PMC3993318

