# Supplementary material for: S-Glutathionylation of human inducible Hsp70 reveals a regulatory mechanism involving the C-terminal α-helical lid
Source: J Biol Chem. 2020 Apr 24;295(24):8302–24. doi: 10.1074/jbc.RA119.012372 (PMC7294093; doi:10.1074/jbc.RA119.012372)
Supplement: Supporting Information [file supp_295_24_8302__index.html]

S-Glutathionylation of human inducible Hsp70 reveals a regulatory mechanism involving the C-terminal α-helical lid — Glutathionylation of human Hsp70 — S-Glutathionylation of human inducible Hsp70 reveals a regulatory mechanism involving the C-terminal α-helical lid — S-Glutathionylation of human inducible Hsp70 — Supporting Information 

# *S*-Glutathionylation of human inducible Hsp70 reveals a regulatory mechanism involving the C-terminal α-helical lid

## Supporting Information

- Supporting Information (to be published online) - Supplementary Figures S1-S6 and Table S1
- Supporting Information (to be published online) - Excel spreadsheet of MS source data for HspA glutathionylation
