# Supplementary material for: S-Glutathionylation of human inducible Hsp70 reveals a regulatory mechanism involving the C-terminal α-helical lid
Source: J Biol Chem. 2020 Apr 24;295(24):8302–24. doi: 10.1074/jbc.RA119.012372 (PMC7294093; doi:10.1074/jbc.RA119.012372)
Supplement: Supporting Information [file supp_RA119.012372_157805_2_supp_517475_q9vn60.pdf]

Supporting Information

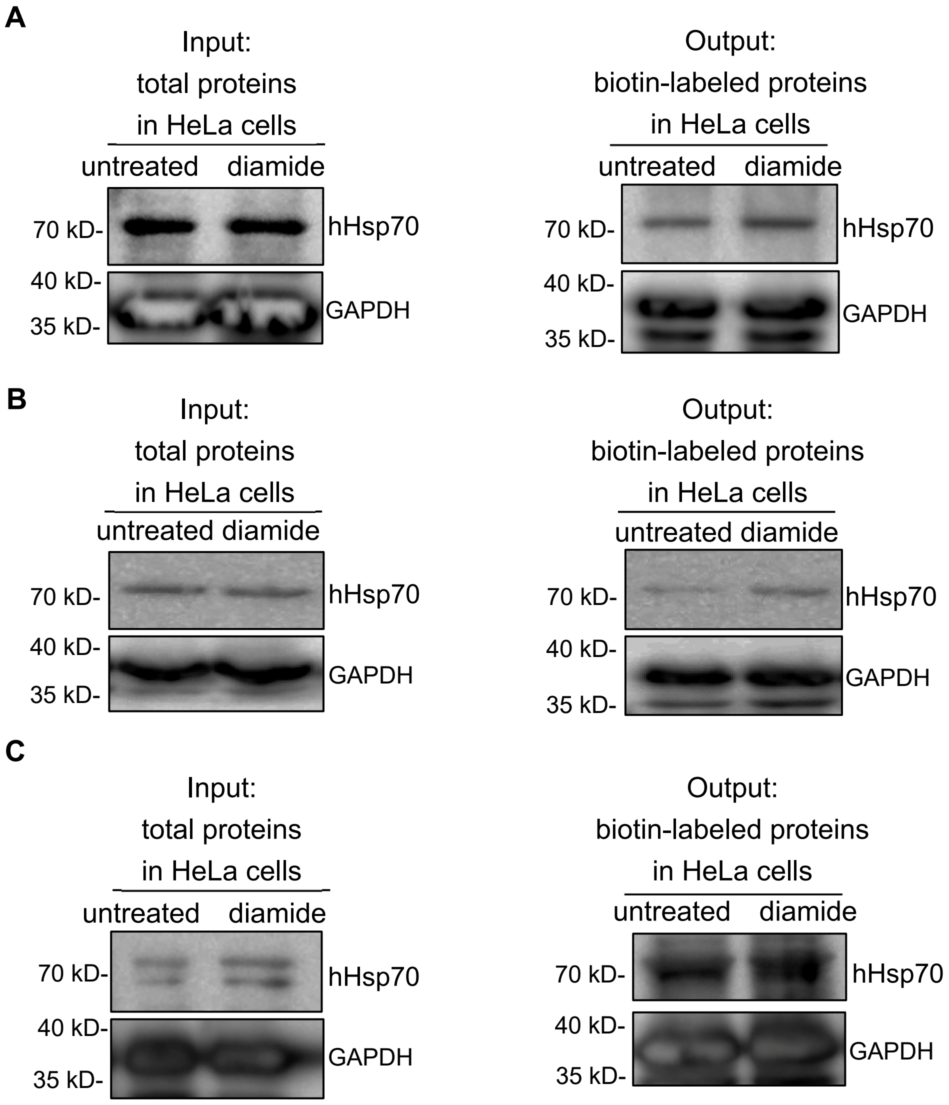

Fig. S1 Western blot detection of glutathionylation of hHsp70 in HeLa cells with or without diamide treatment. The details are as in ***Fig. 1B***. (**A**) 10 mM diamide treatment. (**B-C**) 1 mM diamide treatment.

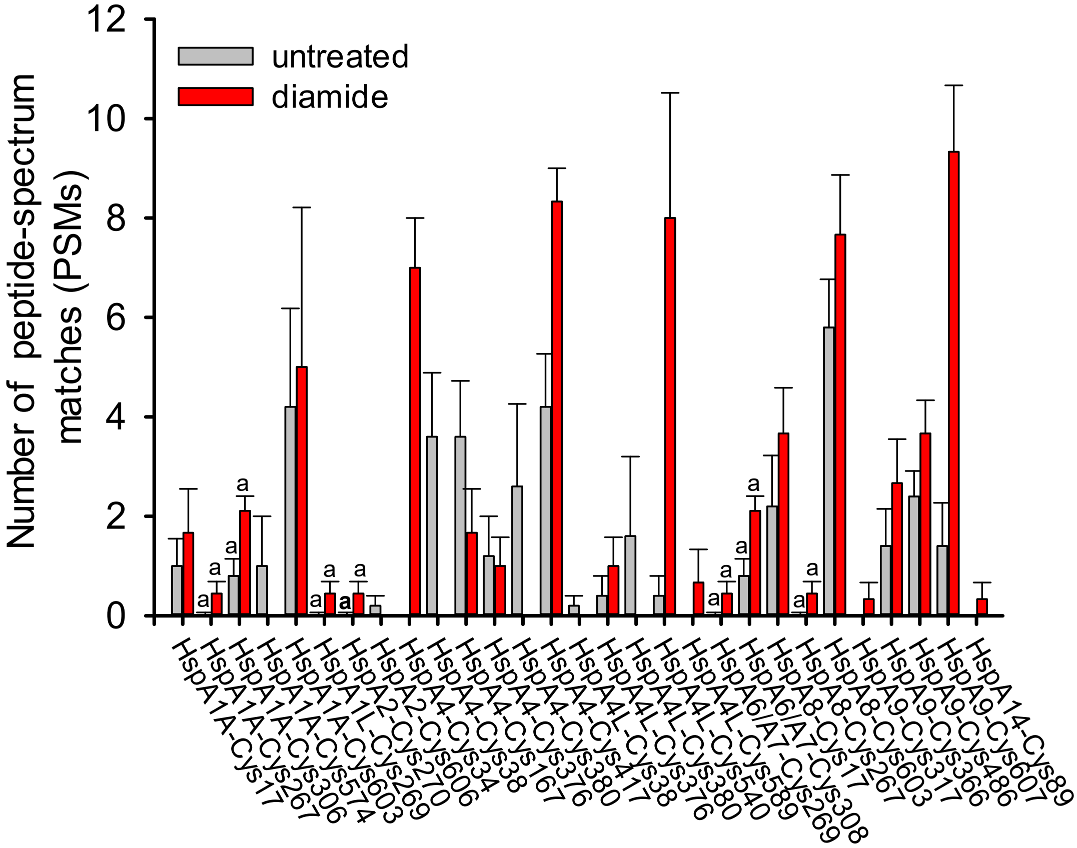

Fig. S2 Summary of glutathionylation sites of Hsp70 family members in HeLa cells with or without diamide treatment detected by mass spectrometry. The details are as in ***Fig. 1C***. The data of untreated samples were averaged and the standard error for five individual MS measurements (***Spreadsheet S1, Data sets 1, 3, 4, 7, 8***) was calculated, and likewise for the data of diamide treated samples (three individual MS measurements, ***Spreadsheet S1, Data sets 2, 5, 6***).

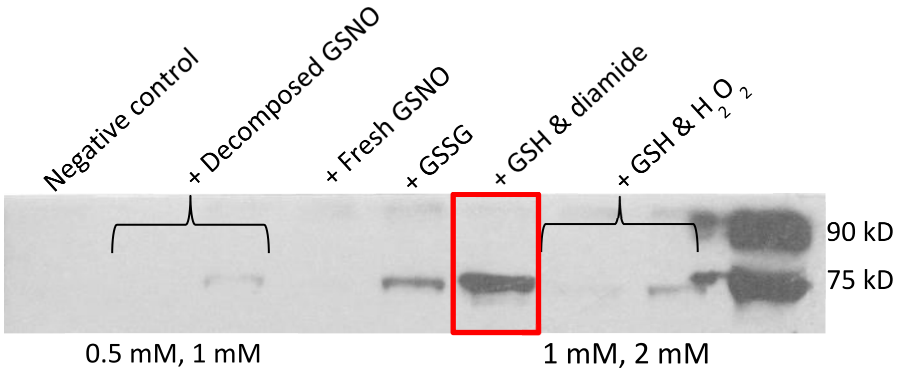

Fig. S3 Western blot detection of glutathionylation of purified hHsp70 under different conditions; 1:500 anti GSH was used in the Western blot detection. Decomposed GSNO of two different concentrations (0.5 mM and 1 mM); fresh GSNO (1 mM); GSSG (1 mM); GSH (1 mM) with diamide (1 mM); or GSH (1 mM) with H_2O_2 (1 mM and 2 mM) were mixed with 15 µM hHsp70 and placed in the dark at 37 °C for 2 h to allow glutathionylation of hHsp70.

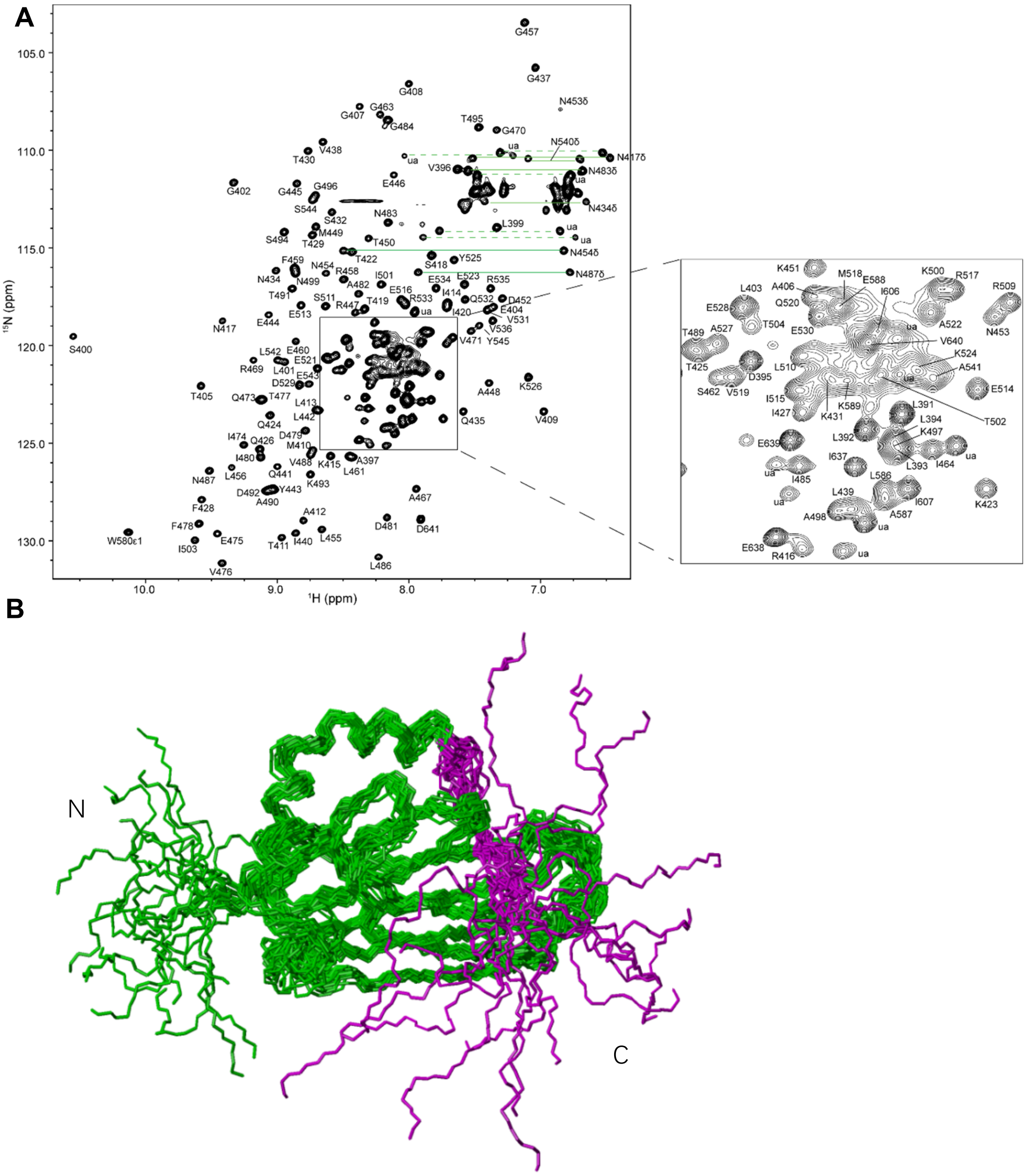

Fig. S4 2D ^{1}H–^{15}N HSQC NMR spectrum at 308 K and the ensemble of conformers of the glutathionylated hHsp70 SBD. (**A**) Assignments are indicated by the one-letter amino acid code and the sequence number (ua, unassigned signals). Nearly complete backbone and side-chain assignments were obtained for residues 390-545, 585-589, 605-607, and 636-641. (**B**) Residues 386-556 are displayed representing the structure of the glutathionylated hHsp70 SBD. The original helix bundle formed by residues 536-610 collapses due to glutathionylation. Residues 386-535 are in green, residues 536-556 are in magenta, and the remaining disordered region (residues 557-641) is not displayed.

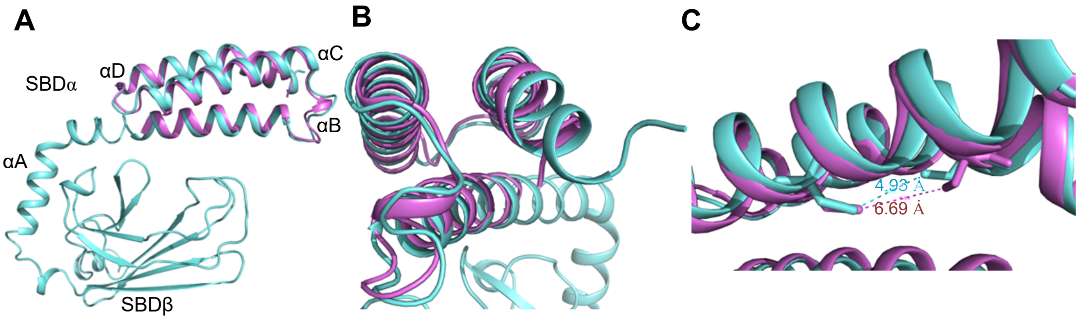

Fig. S5 Structural alignment of isolated hHsp70 SBDα(537-610) (in violet) compared with complete SBD (in blue) indicates influence of SBDβ on the structure of SBDα. (**A**) The crystal structure of the SBD of hHsp70 (PDB code 4PO) and the NMR structure of the isolated SBDα(537-610) of hHs70 (PDB code 2LMG) were aligned. (**B**) The isolated SBDα(537-610) shows a more compact structure than in the complete SBD. (**C**) Distances between C574 and Cys603 were measured in the isolated SBDα(537-610) and in the complete SBD as 4.93 Å and 6.69 Å respectively.

```
sp|A1A766|DNAK_ECOK1            -SGKEQKITIKASSG-LNEDEIQKMVRDAEANAEADRKFEELVQTRNQGDHLLHSTRKQV
sp|P17820|DNAK_BACSU            -TGKEQNITIKSSSG-LSDEEIERMVKEAEENADADAKKKEEIEVRNEADQLVFQTEKTL
sp|P10591|SSA1_YEAST            GTGKSNKITITNDKGRLSKEDIEKMVAEAEKFKEEDEKESQRIASKNQLESIAYSLKNTI
sp|P09446|HSP70A_CAEEL          STGKQNKITITNDKGRLSKDDIERMVNEAEKYKADDEAQKDRIGAKNGLESYAFNLKQTI
tr|A0A1W2WDL2|HSC71IX1_CIOIN    STGKENKITITNDKGRLSKEDIERMVNEAEKYKEEDEKQKEKIQAKNGLESYAFNLKSTV
tr|Q8AVE2|HSC70_XENLA           STGKENKITITNDKGRLSKEDIERMVQEADKYKAEDEKQRDKVSSKNSLESYAFNMKATV
sp|P0DMW0|HSPA1A_RAT            STGKANKITITNDKGRLSKEEIERMVQEAERYKAEDEVQRERVAAKNALESYAFNMKSAV
sp|P0DMV8|HSPA1A_HUMAN          STGKANKITITNDKGRLSKEEIERMVQEAEKYKAEDEVQRERVSAKNALESYAFNMKSAV
sp|P63018|HSPA8_RAT             STGKENKITITNDKGRLSKEDIERMVQEAEKYKAEDEKQRDKVSSKNSLESYAFNMKATV
sp|P11142|HSPA8_HUMAN           STGKENKITITNDKGRLSKEDIERMVQEAEKYKAEDEKQRDKVSSKNSLESYAFNMKATV
                                 :** ::***. ..* *..::*::** :*:     *    : :  :*  :   .. .  :

sp|A1A766|DNAK_ECOK1            EEAG--DKLPADDKTAIESALTALETALKG---EDKAAIEAKMQELAQVSQKLMEIAQQQ
sp|P17820|DNAK_BACSU            KDLE--GKVDEEQVKKANDAKDALKAAIEK---NEFEEIKAKKDELQTIVQELSMKLYEE
sp|P10591|SSA1_YEAST            SEAG--DKLEQADKDTVTKKAEETISWLDSNTTASKEEFDDKLKELQDIANPIMSKLYQA
sp|P09446|HSP70A_CAEEL          EDEKLKDKISPEDKKKIEDKCDEILKWLDSNQTAEKEEFEHQQKDLEGLANPIISKLYQS
tr|A0A1W2WDL2|HSC71IX1_CIOIN    EDDKVKDKISDEDKSAILNKAKEVLDWLENNQTAEKDEYEFQQKELEKIANPIMTKLYQA
tr|Q8AVE2|HSC70_XENLA           EDEKLKGKISDEDKQKILEKCNEVIAWLDKNQTAEKEEFEHQQKELEKVCNPIITKLYQS
sp|P0DMW0|HSPA1A_RAT            EDEGLKGKISEADKKKVLDKCQEVISWLDSNTLAEKEEFVHKREELERVCNPIISGLYQG
sp|P0DMV8|HSPA1A_HUMAN          EDEGLKGKISEADKKKVLDKCQEVISWLDANTLAEKDEFEHKRKELEQVCNPIISGLYQG
sp|P63018|HSPA8_RAT             EDEKLQGKINDEDKQKILDKCNEIISWLDKNQTAEKEEFEHQQKELEKVCNPIITKLYQS
sp|P11142|HSPA8_HUMAN           EDEKLQGKINDEDKQKILDKCNEIINWLDKNQTAEKEEFEHQQKELEKVCNPIITKLYQS
                                .:    .*:   :     .        :.     .      : .:*  : : :     :
```

Fig. S6 The residue Leu542 in the Hsp70 SBDα is conserved from *E. coli* to humans. The red box indicates the Leu residue that will collapse into the SBDβ substrate binding site. Although the sequence position of this Leu residue is 542 in both *E. coli* DnaK and hHsp70, the Leu542 of DnaK is in fact equivalent to Tyr545 of hHsp70 in the sequence alignment and the three-dimensional structures.

Table S1 Primers used for cloning and mutation of proteins used in this study

Protein name	Primers
WT hHsp70 (HspA1A)	Forward, 5'-CATCACGGATCCATGGCCAAAGCCGCGGCAGTC-3'<br>Reverse, 5'-CTAATTAAGCTTATCTACCTCCTCAATGGTGGGGC-3'
hHsp70 SBDα(537-610)	Forward, 5'-GGTCGTGGATCCTCAGCCAAGAACGCCCTG-3'<br>Reverse, 5'-CCTTCTAAGCTTACAGTCCGCTGATGATG-3'
hHsp70 SBD (385-641)	Forward, 5'-GGGGACGGATCCGAGAACGTGCAGGACCT-3'<br>Reverse, 5'-CTAATTAAGCTTATCTACCTCCTCAATGGTGGGGC-3'
hHsp70 C17S	Forward, 5'-CTCCAGCGTGGGGGTGTTCCAACACGGCAAG-3'<br>Reverse, 5'-CACGCTGGAGTAGGTGGTGCCCAGGTCGATG-3'
hHsp70 C267S	Forward, 5'-ACCGCCAGCGAGAGGGCCAAGAGGACCCTG-3'<br>Reverse, 5'-CTCTCGCTGGCGGTGCGCAGCCGCCTCACG-3'
hHsp70 C306S	Forward, 5'-GAGGAGCTGAGCTCCGACCTGTTCCGAAGC-3'<br>Reverse, 5'-AGGTCGGAGCTCAGCTCCTCGAACCTCGCC-3'
hHsp70 C574S	Forward, 5'-GCTGGACAAGAGTCAAGAGGTCATCTCGTGGCTGG-3'<br>Reverse, 5'-CTTGACTCTTGTCCAGCACCTTCTTCTTGTCGGC-3'
hHsp70 C574A	Forward, 5'-GCTGGACAAGGCGCAAGAGGTCATCTCGTGGCTGG-3'<br>Reverse, 5'-CTTGCGCCTTGTCCAGCACCTTCTTCTTGTCGGC-3'
hHsp70 C574Q	Forward, 5'-GCTGGACAAGCAGCAAGAGGTCATCTCGTGGCTGG-3'<br>Reverse, 5'-CTTGCTGCTTGTCCAGCACCTTCTTCTTGTCGGC-3'
hHsp70 C603S	Forward, 5'-GAGCAGGTGAGTAACCCCATCATCAGCGGA-3'<br>Reverse, 5'-CTTGACTCTTGTCCAGCACCTTCTTCTTGTCGGC-3'
hHsp70 C603A	Forward, 5'-GAGCAGGTGGCGAACCCCATCATCAGCGGA-3'<br>Reverse, 5'-ATGGGGTTCGCCACCTGCTCCAGCTCCTTC-3'
hHsp70 C603Q	Forward, 5'-GAGCAGGTGCAGAACCCCATCATCAGCGGA-3'<br>Reverse, 5'-ATGGGGTTCTGCACCTGCTCCAGCTCCTTC-3'
hHsp70 T204A	Forward, 5'-GGGGGCGCCTTCGACGTGTCCATCCTGACGATC-3'<br>Reverse, 5'-TCGAAGGCGCCCCCGCCCAGGTCAAAGATG-3'
WT hHsc70 (HspA8)	Forward, 5'-AAGGAGGGATCCATGTCCAAGGGACCTGCAGTTG-3'<br>Reverse, 5'-TCAACTCGAGTTAATCAACCTCTTCAATGGTGGGCC-3'
WT *E. coli* Grx3	Forward, 5'-CATCACGGATCCATGGCTCAAGAGTTTGTG-3'<br>Reverse, 5'-CCACAAGCTTACTGCAGAGCTCCAATCTGC-3'
E. coli Grx3 C14S	Forward, 5'-CAAAGAAACCTGCCCGTATTCCCATCG-3'<br>Reverse, 5'-GCAGTGCTTTTGCACGATGGGAATACG-3'
E. coli Grx3 C65Y	Forward, 5'-CACAGCACATTGGCGGCTATGATG-3'<br>Reverse, 5'-CAATGCATACAAGTCATCATAGCCG-3'
HSF1	Forward,<br>5'-AAGGAGGGATCCATGGATCTGCCCGTGGGCCCCGGCGC-3'<br>Reverse,<br>5'-TCAGAAGCTTCTAGGAGACAGTGGGGTCCTTGGCTTTGG-3'
